# Design of Neutral Ni[N,N] Catalysts for High-Density Polyethylene Formation: Insights into Catalyst Deactivation

**DOI:** 10.1021/acs.organomet.5c00186

**Published:** 2025-09-26

**Authors:** Bence Szabó, Lennox W. Stewart, Georgina M. Rosair, Stephen M. Mansell

**Affiliations:** Institute of Chemical Sciences, School of Engineering and Physical Sciences, 3120 Heriot-Watt University, Edinburgh EH14 4AS, U.K.

## Abstract

Neutral Ni­[N,O] and cationic Ni­[N,N] complexes are two prominent classes of catalysts for the polymerization of ethylene. We report the amalgamation of these motifs and describe the synthesis and catalytic activity of two neutral Ni­[N,N] catalysts [Ni­(Ph)­(L)­(PPh_3_)], where L = 2-(arylamido)-5-methylcyclopent-2-en-1-arylimine (**7**) or 2-(arylamido)-cyclohex-2-en-1-arylimine (**8**); aryl is the ubiquitous 2,6-diisopropylphenyl group (Dipp). Despite the increased steric bulk, ethylene polymerization was only achieved using B­(C_6_F_5_)_3_ as an activator, forming high-density polyethylene at 0 °C with *T*_m_ up to 140 °C, in contrast to the branched polymers characterized by lower melting points that are more typically formed using nickel catalysts. Surprisingly, both complexes undergo C–H activation over days at room temperature, eliminating benzene and forming a cyclometalated Ni­(II) complex that, for the first time, was characterized by multinuclear NMR spectroscopy and single-crystal X-ray diffraction, structurally characterizing this decomposition route for Ni ethylene polymerization catalysts.

## Graphical abstract

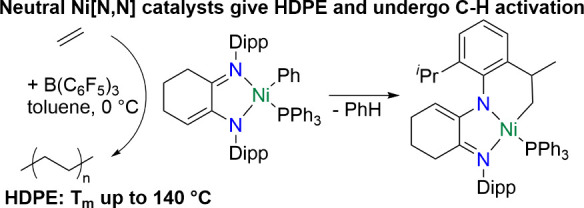

## Introduction

Polyethylene (PE) is the most widely produced polymer globally, accounting for over 34% of nonfiber plastic production,^1^ with an estimated market value of $123.5 billion in 2023.^2^ However, its production contributes to more than 300 million tons of CO_2_ emissions annually,^3^ largely due to the reliance on fossil-fuel-based ethylene derived from energy-intensive processes such as steam cracking. This process produces >140 megatons of ethylene per year^4–6^ but is responsible for 0.7% of the total global emissions of CO_2_.^7^ An emerging strategy to reduce the environmental impact of PE production is the use of bioethanol-derived ethylene.^8–11^ However, this renewable feedstock can contain polar impurities such as water and alcohols, which deactivate the currently utilized oxophilic early transition-metal-based catalysts that are in industrial use.^12,13^ Thus, being able to develop, through deliberate catalyst design, improved water- and alcohol-tolerant catalysts for PE production would allow the use of unpurified ethylene from renewable sources, negating the need for expensive and energy-intensive feedstock purification.

Since the late 1990s, late transition-metal catalysts for PE production based on Ni and Pd have been developed that feature many useful properties.^12,14–16^ Cationic Ni α-diimine catalysts featuring the bulky N-substituent 2,6-diisopropylphenyl (Dipp, **1**; Chart 1) rapidly produce amorphous, branched PE, but are more conveniently generated in situ through the reaction of a precursor with MAO (e.g., **2**).^17–19^ Of specific relevance to this work, Ni allyl complexes similar to **2** that feature a cyclohexene backbone have also been described as catalysts for the production of PE in the patent literature.^20,21^ In contrast to cationic catalysts, neutral Ni catalysts featuring a monoanionic chelating ligand,^22^ which have their origins in the Ni-catalyzed shell higher olefin process (SHOP),^23^ were discovered that did not require activation with MAO and could polymerize ethylene even in the presence of water and polar impurities (**3**).^24^ An activator was often required to remove PPh_3_ and initiate polymerization such as [Ni­(cod)_2_] with **3** (R = H, Ph; cod = 1,5-cyclooctadiene); however, no activator was required if enough steric bulk was present to facilitate PPh_3_ dissociation (**3**; R = anthracenyl).^24^ The related precatalyst **4**, featuring a five-membered chelate ring, polymerized ethylene without an activator (80 °C, 13.8 barg) but had a very short lifetime.^25–27^ The catalyst decomposition product was identified as the homoleptic bis­(anilinotropinato) nickel­(II) complex.^26,27^ The role of the activator is an important one. [Ni­(cod)_2_] was used because it binds strongly to PPh_3_, facilitating the removal of PPh_3_ from the Ni precatalyst.^28–30^ B­(C_6_F_5_)_3_ has been used with the same rationale,^30,31^ although B­(C_6_F_5_)_3_ has also been found to instead react at a remote site present in a precatalyst, forming a B–O bond and resulting in the removal of electron density from Ni in **5**,^32–34^ resembling the cationic Ni centers found in **1** and **2** + MAO.

**Chart 1:**
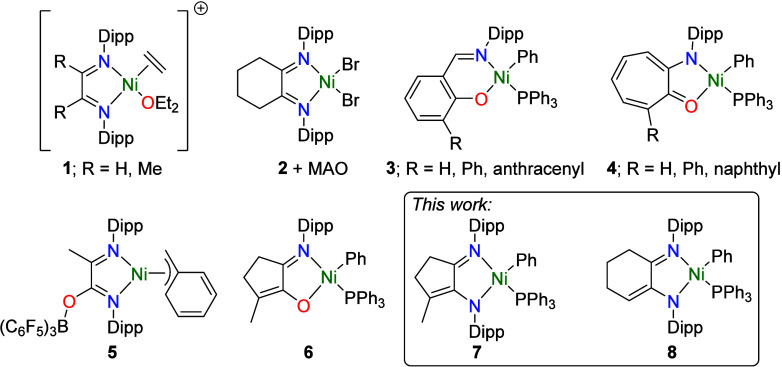
Selected Ni Catalysts for Ethylene Polymerization^a^

a Dipp = 2,6-diisopropylphenyl.

The development of neutral Ni ethylene polymerization catalysts is still a burgeoning field,^31,35–38^ especially with a view to producing new catalysts that are more compatible with future sustainable streams of ethylene. Previous work in our group showed that precatalyst **6**, which features a ligand based on maple lactone,^39^ is active for ethylene polymerization only when used with an activator at higher temperatures, with the highest activity at 80 °C.^40^ However, activation with [B­(C_6_F_5_)_3_] or [Ni­(cod)_2_] produced very different PE architectures, giving PE with either a moderate *T*_m_ (≈117 °C) or low *T*_m_ (≈51 °C), respectively. We identified great potential for this ligand system to finally achieve the direct comparison of a neutral Ni­[N,N] catalyst, featuring two of the ubiquitous Dipp groups (**7**), with an analogous neutral Ni­[N,O] catalyst (**6**) and area-defining cationic Ni­[N,N] catalysts, such as **1**. This would ultimately help unpick the competing factors that are required to generate successful catalysts and therefore increase our understanding of nickel catalyst design in order to develop catalysts that achieve three goals: high activity, the production of PE with the sought-after properties (e.g., HD-PE featuring a high *T*_m_), and tolerance to water/ethanol/polar monomers. In addition, the analogous complex **8**, featuring a cyclohexene backbone,^17,41^ was found to be more easily synthesized than the maple-lactone analogue, allowing us to cast new light on the differences between closely related catalyst systems that are derived from different precatalysts/activators. We were also able to structurally identify a long speculated-upon decomposition product.^42^

## Results and Discussion

### Formation and Deprotonation of Proligands

The imino-enamine proligand **9** was synthesized as described in the literature^17^ from 1,2-cyclohexanedione (drawn as its energetically preferred keto–enol tautomer)^43^ and H_2_NDipp at room temperature (Scheme 1). In great contrast, the analogous proligand **12**, which is a new compound, required temperatures of 140 °C for 4 days for the first condensation step to produce the previously described keto-enamine **11** ^40^ and then 170 °C for 4 days for the second condensation step to give imino-enamine **12** (Scheme 1). Combining the two steps is possible as long as the maple lactone is not allowed to sublime out of the reaction mixture in the first step. Deprotonation to form the sodium salts did not go to completion using NaN­(SiMe_3_)_2_ as the base; however, this was remedied by using the stronger base NaCH_2_Ph, which is convenient to handle, goes into solution as it reacts, giving a visual indication of the reaction progress, and eliminates toluene as the only byproduct. Multinuclear NMR spectroscopy and single-crystal X-ray diffraction experiments established the structures of **10**, **12**, and **13** (Figures 1 and S25), with the C=N double bond of the imine identified by its shorter bond length and the enamide (enamine for **12**) identified by the longer C–N bond length and the presence of the conjugated C=C double bond (see the ESI for details). The sodium salts **10** and **13** are dimeric in the solid state, with the sodium cations showing contacts to an arene ring of another ligand.

**Scheme 1:**
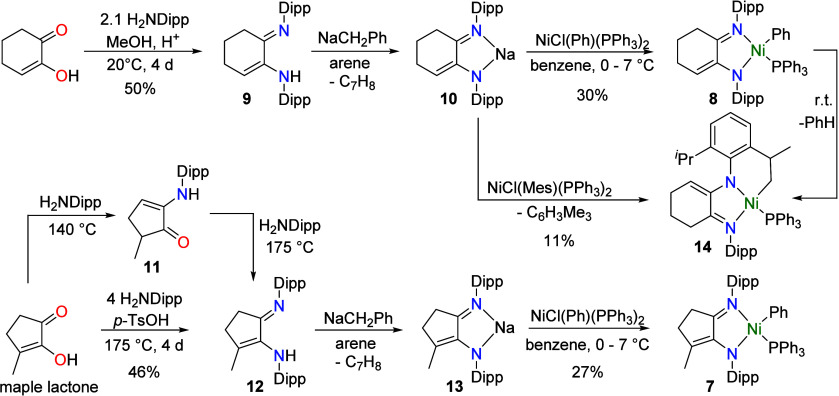
Synthesis of Proligands and Nickel Complexes

**Figure 1:**
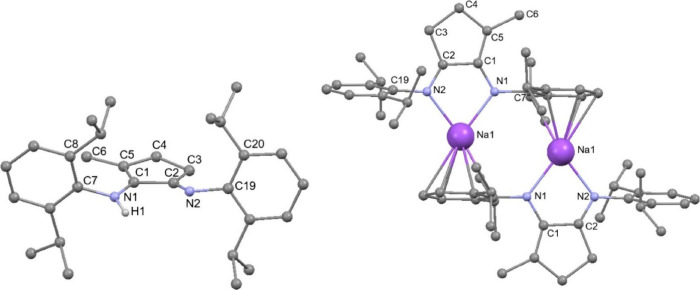
Molecular structure of **12** (left) and **13** (right). All H atoms, except those on nitrogen, have been omitted for clarity. Na···arene contacts vary from 2.761(2) to 2.873(2) Å.

### Synthesis of Nickel Complexes

The synthesis of targeted nickel precatalysts proved to be very challenging. Although previously we established that reactions of the sodium salt of **11** with *trans*-[Ni­(Ph)­(Cl)­(PPh_3_)_2_][Bibr ref44] proceeded without issue to form **6** (Chart 1),^40^ the reaction of the sodium salts of imino-enamides **10** and **13** with *trans*-[Ni­(Ph)­(Cl)­(PPh_3_)_2_] gave rise to several products that were identified as [Ni­(PPh_3_)_3_] (by single-crystal X-ray diffraction experiments and comparison of ^31^P­{^1^H} NMR spectra with literature reports), reformed protonated ligands **9** and **12**, as well as the expected PPh_3_ and the new catalysts **7** and **8** (Scheme 1). Extensive optimization of these reactions established that trace water was not present and was not the cause of **9** and **12**, that using the lithium and potassium salts of the proligands produced poorer results, and that the low stability^45^ of *trans*-[Ni­(Ph)­(Cl)­(PPh_3_)_2_] or other intermediates on route to **7** and **8** was now a prominent issue, whereas previously it had not presented a problem in the synthesis of **6**.^40^ Optimization of the reaction conditions showed that using benzene as the solvent resulted in better yields than toluene and that the reaction was best conducted when cold for the minimum amount of time. Two recrystallizations from alkane solvents gave pure complexes but in low yields of around 30%. Characterization of the structures of **7** and **8** was achieved using multinuclear NMR spectroscopy and single-crystal X-ray diffraction. Four doublets were evident for both **7** and **8** from the isopropyl groups together with two pseudo-septets, which were very broad for **8**. The alkene proton in **8** was evident as a triplet at 4.73 ppm, and three protons in the Ni-Ph group formed an overlapping multiplet at around 6.2 ppm. ^31^P­{^1^H} NMR singlets were observed at 14.2 (**7**) and 13.9 ppm (**8**). X-ray diffraction revealed distorted square planar geometries for the four-coordinate Ni centers (Figure 2). The P atom is situated out of the plane formed by the Ni­[N,N] atoms (0.65 Å for **7**, 0.60 Å for **8**), while the Ni–C bond is maintained in this plane, although the Ph group is orientated approximately perpendicular to it. This distortion away from square planar provides evidence of the large amount of steric crowding in these complexes, particularly between the PPh_3_ and the Dipp groups. For **7**, the location of the imine and enamide functionalities was obvious, facilitated, no doubt, by the methyl substituent that generates a more substituted alkene which helps fix the double bond in this location. However, the key Ni–N, C–C, and C–N bond lengths in **8** were identical within error, and therefore, these moieties were not differentiated in the solid state, leading to the conclusion that the structure is disordered (for clarity, only one Lewis structure of **8** is shown in Scheme 1 and only one position of the disordered cyclohexene ring in Figure 2).

**Figure 2:**
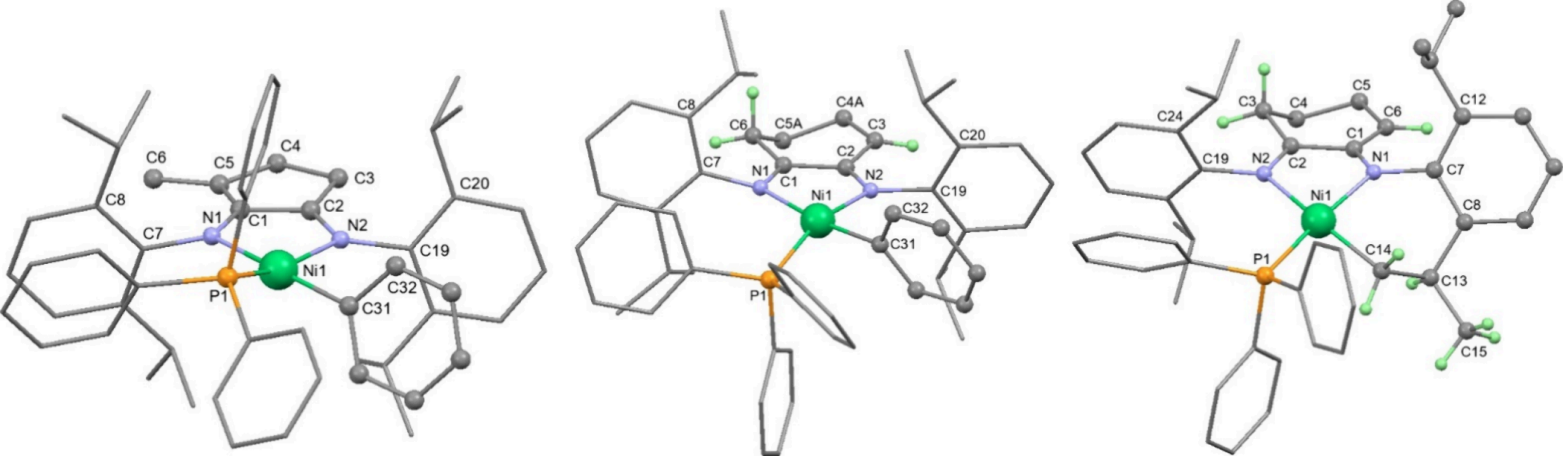
Molecular structures of **7** (LHS), **8** (middle), and **14** (RHS). Only significant H atoms have been labeled for clarity. For **8**, only one position of the backbone carbon atoms and ligand H atoms has been shown (0.5 occupancy).

Neither **7** nor **8** is stable indefinitely at room temperature in solution. For **7**, slow decomposition at room temperature is evident over several weeks with two new phosphorus-containing products observed by ^31^P­{^1^H} NMR spectroscopy in a ratio of about 1:1. Evidence that these products are highly asymmetric is provided by the large number of doublets arising from the isopropyl methyl groups in the ^1^H NMR spectra (Figure S19). In addition, two new characteristic multiplet resonances, also in a ratio of about 1:1, were observed near 0 ppm. For **8**, similar results were obtained, albeit more quickly (18 h at room temperature), with two phosphorus products formed in a ratio of approximately 1:1.7 and two similar multiplets around 0 ppm observed in the ^1^H NMR spectra. The nature of one of these decomposition products became evident only when the synthesis of a Ni-mesityl complex was attempted. Reaction of **10** with [Ni­(Mes)­(Cl)­(PPh_3_)_2_] (Mes = 2,4,6-Me_3_C_6_H_2_) gave a low yield of the cyclometalated product **14** after recrystallization (Figure 1, RHS), whose NMR spectra exactly matched one of the decomposition products of **8** (see Figure S24). X-ray diffraction was essential in unambiguously demonstrating that C–H activation of the enamide Dipp substituent at a methyl group had occurred, resulting in loss of arene (either mesitylene or benzene for the onward reaction of **7**/**8**) and formation of a 6-membered metallacycle. The complexity of the ^1^H NMR spectra could now be understood with H14A and H14B assigned using the Karplus relation for the dihedral angles with respect to H13 as coupling was only seen in the 2D COSY NMR spectrum for the axial disposition between H13 and H14A (Figure S28). The NMR spectrum of the product formed from the Ni-mesityl complex (**14**) exactly matches one of the decomposition products from **8**. Therefore, C–H activation and cyclometalation lead to one of the decomposition products of **8**, with an analogous reaction also likely to be occurring for **7** based on the similarity of the ^1^H NMR spectra, both chemical shifts and coupling of the diagnostic resonances.

Comparing this reaction and the structure of **14** to literature examples shows several differences from what has been established for Ni­[N,N] complexes previously. Interestingly, the diethyl analogue of **2** (Chart 1), synthesized from the reaction of **2** with 2 EtMgCl, has been characterized as the 1,2-bis­(imino)­cyclohexane diethyl nickel­(II) complex, which undergoes protonation to produce a structurally characterized cationic Ni-Et complex, featuring a β-agostic interaction; no C–H activation was observed.^46^ This is similar to a neutral nickel β-diketiminate ethyl complex^47^ and a neutral nickel anilinotropiminate ethyl complex^27^ that also formed a β-agostic interaction rather than resulting in C–H activation of an aryl substituent. Cyclometalation of an *ortho*-methyl substituent was observed in the reaction of the lithium salt of an aniline-oxazoline proligand with *trans*-[Ni­(Ph)­(Cl)­(PPh_3_)_2_], which formed directly without a Ni-phenyl intermediate being observed.^48^ C–H activation of the Dipp group of a cationic Pd α-diimine complex has been described; however, the resulting cyclometalated complex was not isolated.^42^ C–H activation of a Dipp isopropyl group is a competing pathway in the decomposition of a cyclopentadienyl N-heterocyclic carbene Rh­(I) complex that was recently shown instead to undergo reversible C–C bond activation at the Ar-*^i^* Pr bond.^49^

### Polymerization of Ethylene

The polymerization of ethylene was then screened to see how the catalyst design affected the reactivity with ethylene and the polymer properties. Our hypothesis that the additional steric bulk of a second Dipp group would facilitate PPh_3_ dissociation and remove the need for an activator was shown to be incorrect as neither **7** nor **8** would polymerize ethylene by themselves at 25 or 70 °C under 10 barg ethylene (Table 1). However, the addition of B­(C_6_F_5_)_3_ gave PE for both **7** and **8**. What was most interesting was the nature of the PE formed. Differential scanning calorimetry (DSC) showed a melting transition of 138–140 °C for the PE formed when the polymerization was carried out at 0 °C for **7** and **8**, which corresponds to HD-PE.^17^ DSC investigations of these melted polymer samples showed a lower *T*_m_ for the second cycle (129–134 °C). The formation of solid HD-PE for **8** had completely halted the reaction before the reaction had been quenched, and further optimization of the conditions would be required to establish meaningful activities for these catalysts. Increasing the polymerization temperature for **8** gave PE with a lower melting point: 115 °C for the reaction run at 25 and 55 °C for the reaction run at 70 °C. High-temperature GPC analysis of the polymers produced by **8** showed very high number-average molecular weights (*M*_n_) for the polymers produced at 0 and 25 °C of around 390,000 g mol^–1^ (Table 1). High^24,27^ and even ultrahigh^50^ molecular-weight PE can be produced by other neutral Ni catalysts when the conditions and catalyst are carefully selected. For example, catalyst **4** (Chart 1) without additional activator gave high *M*_n_ PE of 82,000–530,000 g mol^–1^ across a range of reaction temperatures (40–60 °C) and pressures of ethylene (3.4–55 barg).^27^ *M*_n_ decreased to 45,000 g mol^–1^ for the polymer produced by **8** at 70 °C, and the dispersity ranged from 2.1 to 3.2, values which are similar to the PE produced by **6** at 60 °C (*M*_n_ up to 38,000 g mol^–1^ with [Ni­(cod)_2_] as thecocatalyst); **6** had very low activity at 25 °C and so low-temperature comparisons cannot be made.^40^

**Table 1:** Polymerization of Ethylene with Precatalysts **7** and **8** ^a^

entry	catalyst	temperature/°C	activator	ethylene pressure/barg	mass of the polymer/g	activity/kg (mol h bar)^−1^	*M*_w_ (g mol^–1^)	*M*_n_ (g mol^–1^)	*Đ*	*T*_m_/°C^d^ first cycle	*T*_m_/°C^d^ second cycle
1	**7**	25	none	10	0	0					
2	**7**	70	none	10	0	0					
3^b^ ^,^ ^c^	**7**	0	B(C_6_F_5_)_3_	10	0.029	0.48	n.d.	n.d.	n.d.	138	134
4	**8**	25	none	10	0	0					
5	**8**	70	none	10	0	0					
6^b^	**8**	0	B(C_6_F_5_)_3_	10	1.459	12.1	1,247,731	391,235	3.2	140	129
7	**8**	25	B(C_6_F_5_)_3_	10	0.635	10.5	810,022	388,453	2.1	115	96
8	**8**	70	B(C_6_F_5_)_3_	10	0.270	4.47	105,056	44,927	2.3	55	n.d.

a Autoclave was preheated or cooled to the indicated temperature, before loading a solution of the catalyst and activator in toluene (30 mL). The reaction was quenched with acetone after 30 min.

b The reaction was run for 60 min.

c The precatalyst **7** for this run only contained a small amount of free ligand (**12**) as an impurity.

d DSC was used to obtain melting transitions; first cycle was from the sample as formed. Second cycle was the same polymer from the first cycle, which had melted and then crystallized. GPC data for entry 3 was not determined (n.d.) due to the small amount of polymer obtained. *Đ* = dispersity = *M*_w_/*M*_n_.

For comparison, **2** + MAO under 13.8 barg ethylene at 35 °C gave PE with a *T*_m_ of 100 °C and an *M*_n_ of 844,000 g mol^–1^;^17^ however, it is unknown what tautomer the ligand is in for the actual active species, and therefore whether this system is a traditional cationic Ni­[N,N] catalyst, or whether deprotonation of the ligand had occurred in situ. Increasing the temperature to 60 and 80 °C lowered the *T*_m_ to 57 and 20 °C, respectively, and *M*_n_ to 395,000 and 210,000 g mol^–1^, respectively, as seen above for **8**, with activity also decreasing as a result of catalyst decay.^17^ In fact, it was stated that most of the catalyst had deactivated after 10 min at 85 °C.^17^ Comparing Ni­[N,N] catalysts **7** and **8** with analogous Ni­[N,O] catalysts shows a dramatic change in the nature of the PE produced. **6** produced PE with a *T*_m_ of 117 or 51 °C depending on the activator B­(C_6_F_5_)_3_ or [Ni­(cod)_2_], respectively. **4** gave PE with a higher degree of branching as the reaction temperature was increased through the classic chain walking mechanism.^27^

## Conclusions

Two Ni­[N,N] catalysts featuring two N-Dipp substituents have been synthesized, despite the challenge of multiple reaction pathways being accessible in the reaction of the sodium salts of the proligands with *trans*-[Ni­(Ph)­(Cl)­(PPh_3_)_2_]. Both complexes decompose in solution at room temperature over days to yield a C–H activated, cyclometalated product arising from deprotonation of a Dipp methyl group with loss of arene. Despite the substantial increase in steric bulk, neither Ni­[N,N] complex would polymerize ethylene by itself; however, the addition of the activator B­(C_6_F_5_)_3_ gave an active catalyst that produced HD-PE at low temperatures (0 °C). The presence of two N-Dipp groups therefore produced a catalyst that works at lower temperatures and gives more crystalline PE than the analogous Ni­[N,O] complex, which was active only at higher temperatures and gave PE with a lower melting transition. The interplay between neutral and monoanionic ligands in Ni catalysis and controlling the reactivity of the ubiquitous Dipp substituent is therefore the subject of our continuing interest.

## Experimental Section

All reactions requiring inert conditions were performed under an oxygen-free nitrogen atmosphere by using standard Schlenk line techniques or by using an MBRAUN UNILab Plus glovebox, unless otherwise noted. Dry toluene, DMF and THF were obtained from a solvent purification system (MBraun SP-300) and stored over 4 Å molecular sieves prior to use. Benzene was dried over molten potassium and distilled or dried over activated 4 Å molecular sieves prior to use. Nondry solvents were used as received from Fisher Scientific. 2,6-Diisopropylaniline was distilled under a vacuum before use. The following starting materials were made using established procedures: **9**,^17^ sodium benzyl,^51^ *trans*-[Ni­(Ph)­(Cl)­(PPh_3_)_2_],^44^ and *trans*-[NiCl­(mesityl)­(PPh_3_)_2_].^52^ B­(C_6_F_5_)_3_ was a gift from Prof. M. Ingleson (University of Edinburgh) and featured no bound water.

NMR spectra were obtained using either an AVIII400 (400 MHz) or AVIIIHD (400 MHz) spectrometer. ^1^H NMR spectra were recorded at 400 MHz and referenced to the residual solvent peak (7.24 for CDCl_3_, 2.09 for toluene-d_8_, and 7.16 for C_6_D_6_). ^13^C­{^1^H} NMR spectra were recorded at 101 MHz and referenced to the residual solvent peak (77.16 ppm for CDCl_3_ and 128.06 ppm for C_6_D_6_). FTIR spectroscopy was performed on a Thermo Scientific Nicolet iS5/iD5 ATR spectrometer. Elemental analyses were performed at Heriot-Watt University (non-air-sensitive samples only). Purity for air-sensitive complexes was established using multinuclear NMR spectroscopy. DSC measurements were carried out on a TA Instruments DSC 2010.

### Safety Statement

Benzene is an irritant and a highly flammable liquid that may cause genetic defects, may cause cancer, or may cause damage to organs by repeated exposure. As such, it should be handled with care and appropriate safety precautions.
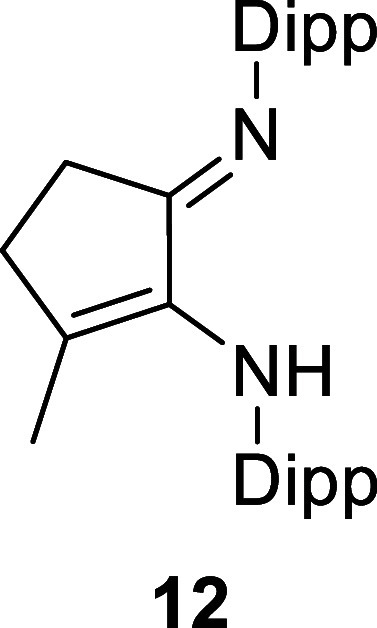


### Two-Step Synthesis

Maple lactone (3.650 g, 32.6 mmol, 1 equiv) was suspended in a mixture of xylenes (20 mL) and 2,6-diisopropylaniline (11.6 g, 65.2 mmol, 2 equiv) in a round-bottom flask. *para*-Toluenesulfonic acid monohydrate (18.5 mg, 0.100 mmol, 0.003 equiv) was added, a Dean–Stark apparatus and waterless condenser were attached, and the mixture was heated to reflux for 3 days at 140 °C in an oil bath. After 3 days, the pale brown solution that was formed was concentrated under reduced pressure to remove xylene and then was fractionally distilled under vacuum. The monocondensation product (**11**) was collected at 130 °C. To the collected yellow oil (**11**), 2,6-diisopropylaniline (11.6 g, 65.2 mmol, 2 equiv) and *para*-toluenesulfonic acid (19.0 mg, 0.100 mmol, 0.003 equiv) were added, and the mixture was heated under nitrogen for 4 days at 170 °C. The obtained black, dense oil was purified by column chromatography (1% ethanol in petroleum ether 40–60). Recrystallization from hot methanol yielded an off-white crystalline product (4.073 g, 29%).

### One-Step Synthesis

Maple lactone (3.650 g, 32.6 mmol, 1 equiv) and 2,6-diisopropylaniline (23.2 g, 130.4 mmol, 4 equiv) were reacted at 170 °C, without adding the solvent, in the presence of *para*-toluenesulfonic acid monohydrate (19.0 mg, 0.100 mmol, 0.003 equiv). After 4 days, the same purification procedure was carried out as for the two-step synthesis, yielding **12** (6.88 g, 49%).

^1^H NMR (400.1 MHz, 298 K, C_6_D_6_) δ/ppm: 7.25–7.21 (m, 2H, Ar-*H*), 7.21–7.14 (m, 2H, Ar-*H*), 7.13–7.10 (m, 2H, Ar-*H*), 6.08 (s, 1H, N*H*), 3.55 (hept, *J* = 6.8 Hz, 2H, 2 × *^i^* Pr-*H*), 3.14 (hept, *J* = 6.8 Hz, 2H, 2 × *^i^* Pr-*H*), 2.08–2.02 (m, 2H, C*H*_*2*_), 2.00–1.96 (m, 2H, C*H*_*2*_), 1.31–1.26 (m, 9H, 2 × *^i^* Pr–C*H*_*3*_ overlapping with C=C–C*H*_*3*_), 1.26–1.18 (m, 18H, 6 × *^i^* Pr–C*H*_*3*_); ^13^C­{^1^H} NMR (100.6 MHz, 298 K, C_6_D_6_) δ/ppm: 173.23 (C), 148.00 (C), 147.37 (C), 138.62 (C), 137.44 (C), 136.98 (C), 126.94 (CH), 125.19 (C), 123.34 (CH), 123.16 (CH), 123.14 (CH), 32.29 (CH_2_), 28.62 (CH_3_), 28.30 (CH_3_), 27.10 (CH_2_), 24.72 (CH_3_), 23.67 (CH_3_), 23.25 (CH_3_), 22.92 (CH_3_), 14.64 (CH_3_); Elemental analysis: Calcd (%) for C_30_H_42_N_2_: C, 83.67; H, 9.83; N, 6.50. Found: C, 83.32; H, 9.90; N, 6.60.
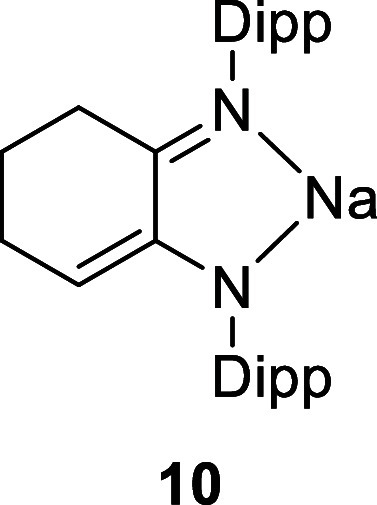


**9** (43.1 mg, 0.100 mmol, 1 equiv) and sodium benzyl (11.4 mg, 0.100 mmol, 1 equiv) were dissolved in benzene solvent (10 mL) and agitated until a homogeneous solution was formed. In further syntheses, the ligand salt was used in situ without the removal of the solvent.

^1^H NMR (400.1 MHz, 298 K, C_6_D_6_) δ/ppm: 7.18 (m, 2H, Ar-*H*), 7.08 (dd, *J* = 7.6, 1.7 Hz, 3H, Ar-*H*), 6.48 (t, *J* = 7.5 Hz, 1H, Ar-*H*), 4.16 (t, *J* = 4.4 Hz, 1H, C=C*H*), 3.58 (hept, *J* = 6.7, 6.2 Hz, 2H, *^i^* Pr-*H*), 2.83 (hept, *J* = 6.7 Hz, 2H, *^i^* Pr-*H*), 2.25 (q, *J* = 5.7 Hz, 2H, C*H*_*2*_), 1.56 (m, 2H, C*H*_*2*_), 1.30 (dd, *J* = 13.3, 6.9 Hz, 18H, *^i^* Pr–C*H*_*3*_), 1.13 (d, *J* = 6.9 Hz, 6H, *^i^* Pr–C*H*_*3*_); ^13^C­{^1^H} NMR (100.6 MHz, 298 K, C_6_D_6_) δ/ppm: 174.46 (C), 157.76­(C), 149.70­(C), 146.71­(C), 146.26­(C), 137.55­(C), 124.23 (CH), 123.69 (CH), 123.56 (CH), 118.91­(CH), 100.44 C=CH, 32.31 (CH_2_), 28.44 (CH_3_), 28.04 (CH_3_), 26.94 (CH_2_), 25.29 (CH_3_), 25.04 (CH_3_), 24.78 (CH_2_), 24.43 (CH_3_), 23.34 (CH_3_).
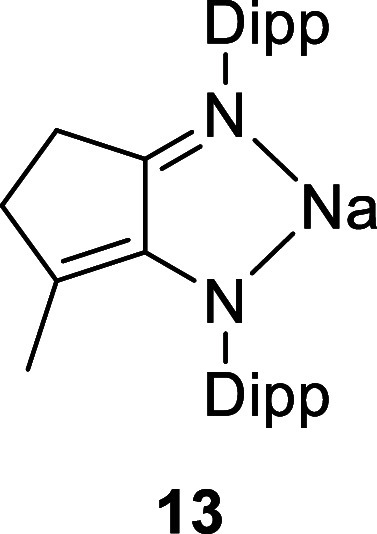


**12** (40.6 mg, 0.09 mmol, 1 equiv) and sodium benzyl (11 mg, 0.09 mmol, 1 equiv) were dissolved in toluene or benzene (0.7 mL). After 3 min, all NaCH_2_Ph had reacted, giving a clear yellow solution. In further syntheses, the ligand salt was used in situ without removal of the solvent.

^1^H NMR (400.1 MHz, 298 K, C_6_D_6_) δ/ppm: 7.20 (m, 2H, Ar-*H*), 7.11 (s, 1H, Ar-*H*), 7.06 (m, 2H, Ar-*H*), 6.58 (t, *J* = 7.5 Hz, 1H, Ar-*H*), 3.62 (hept, *J* = 6.9 Hz, 2H, 2 × *^i^* Pr–C*H*), 2.93 (hept, *J* = 6.9 Hz, 2H, 2 × *^i^* Pr–C*H*), 2.16 (m, 2H, C*H*_*2*_), 2.05 (m, 2H, C*H*_*2*_), 1.33 (d, *J* = 6.9 Hz, 9H, 2 × *^i^* Pr–C*H*_*3*_ overlapping with C*H*_*3*_), 1.27 (dd, *J* = 6.9, 1.9 Hz, 12H, 4 × *^i^* Pr–C*H*_*3*_), 1.14 (d, *J* = 6.9 Hz, 6H, 2 × *^i^* Pr–C*H*_*3*_); ^13^C­{^1^H} NMR (100.6 MHz, 298 K, C_6_D_6_) δ/ppm: 185.6 (s, *C*=N), 158.7 (s, Ar C), 148.42 (d, *J*_C–P_ = 13.4 Hz,), 144.3 (s, Ar C), 138.0 (s, Ar C), 124.2 (s, Ar CH), 123.7 (s, Ar CH), 122.8 (s, Ar CH), 118.5 (s, Ar CH), 117.9 (s, Ar C), 32.4 (s, CH_2_), 28.8 (s, CH_2_), 28.4 (s, *^i^* Pr CH), 24.8 (s, *^i^* Pr Me), 24.6 (s, *^i^* Pr Me), 24.0 (s, *^i^* Pr Me), 23.1 (s, *^i^* Pr Me), 17.8 (s, backbone Me).
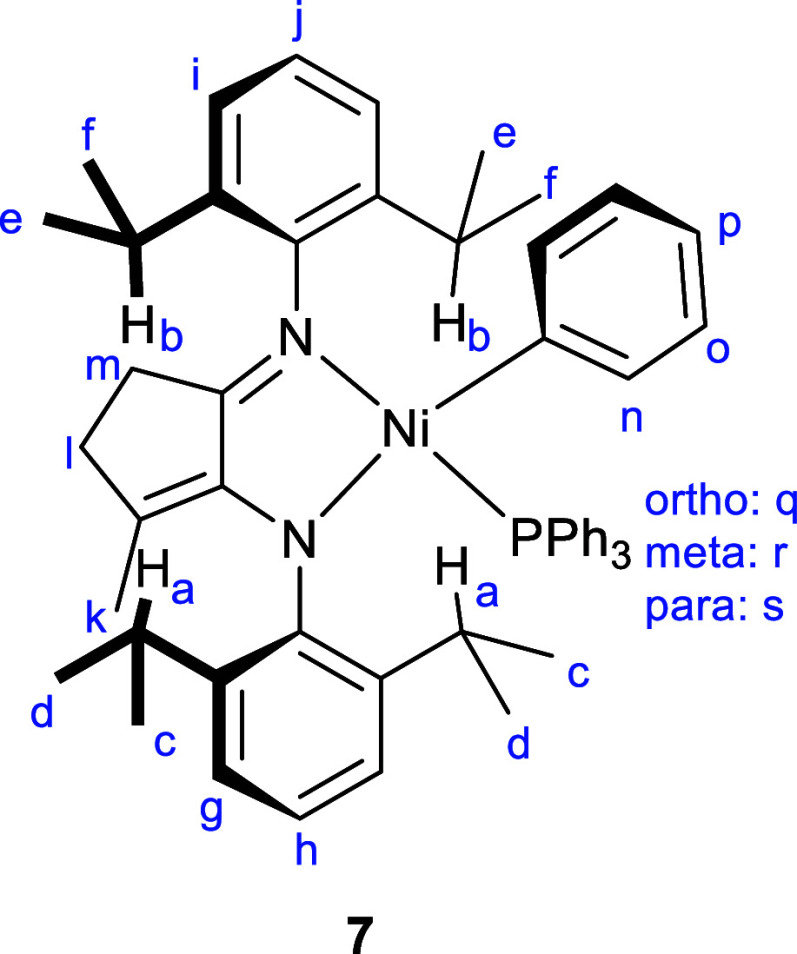


**12** (219 mg, 0.500 mmol 1 equiv) and sodium benzyl (57.0 mg, 0.500 mmol, 1 equiv) were dissolved in benzene (10 mL) and were heated to 50 °C for 1 h. The formed yellow solution was cooled in an ice bath, allowing the mixture to solidify. *trans*-[NiCl­(Ph)­(PPh_3_)_2_] was added as a powder. The ice bath was then replaced with a cold-water bath at 7 °C, allowing the solidified solution to melt and mix with the nickel complex slowly. After 1 h, the solvent was evaporated, and the resulting dark brown oil was recrystallized from cold hexane. A second recrystallization from pentane yielded small, plate-shaped dark orange crystals of **7** (110 mg, 27%).

^1^H (400.1 MHz, 298 K, C_6_D_6_) δ/ppm: 7.62 (m, 6 H, ortho-PPh_3_), 7.41 (bs, 2 H, m-Dipp), 7.10–6.98 (m, 8 H, Ar), 6.93–6.86 (m, 11 H, overlapping Ar and ortho-Ph), 6.26–6.17 (m, 3 H, m and p-Ph), 4.28 (pseudo-septet, 2 H,^3^ *J*_H–H_ = 6.6 Hz, H_a_), 3.95 (pseudo-septet, 2 H,^3^ *J*_H–H_ = 6.8 Hz, H_b_), 2.15 (m, 2 H, CH_l,2_), 2.01 (m, 2 H, CH_m,2_), 1.34 (d, 6 H,^3^ *J*_H–H_ = 6.8 Hz, Me_d_), 1.24 (d, 6 H,^3^ *J*_H–H_ = 6.8 Hz, Me_e_), 1.14 (d, 6 H,^3^ *J*_H–H_ = 6.8 Hz, Me_c_), 1.13 (s, 3 H, Me_k_ on 5 m.r.), 1.05 (d, 6 H,^3^ *J*_H–H_ = 6.8 Hz, Me_f_); ^13^C­{^1^H} (100.6 MHz, 298 K, C_6_D_6_) δ/ppm: 192.3 (s, *C*=N), 156.4 (d, *J*_C–P_ = 3.0 Hz, C=*C*-N), 146.9 (s, 4° C), 146.1 (d, *J*_C–P_ = 51.9 Hz, ipso-Ni-Ph), 141.2 (s), 136.6 (d, *J*_C–P_ = 4.4 Hz, Ni-Ph), 134.8 (d, *J*_C–P_ = 10.7 Hz, *ortho*-PPh_3_), 134.5 (d, *J*_C–P_ = 11.2 Hz, ipso-PPh_3_), 129.4 (d, *J*_C–P_ = 1.9 Hz, *para*-PPh_3_), 127.5 (d, *J*_C–P_ = 9.7 Hz, *meta*-PPh_3_), 126.2 (s), 125.1 (narrow doublet, *J*_C–P_ = 1.5 Hz, m-Ph-Ni), 124.5 (s, 4° Ar), 123.9 (s, Ar CH), 123.7 (s, Ar CH), 123.0 (s, Ar CH), 121.3 (s, p-Ph-Ni), 36.9 (s, CH_2_, C_l_), 28.81 (s, *^i^* Pr *C*H), 28.76 (s, *^i^* Pr *C*H), 27.7 (s, CH_2_, C_m_), 25.3 (s, *^i^* Pr Me), 25.1 (s, *^i^* Pr Me), 24.1 (s, *^i^* Pr Me), 24.0 (s, *^i^* Pr Me), 22.8 (s), 17.0 (s, backbone Me); ^31^P­{^1^H} NMR (162 MHz, 298 K, C_6_D_6_) δ/ppm: 14.2.
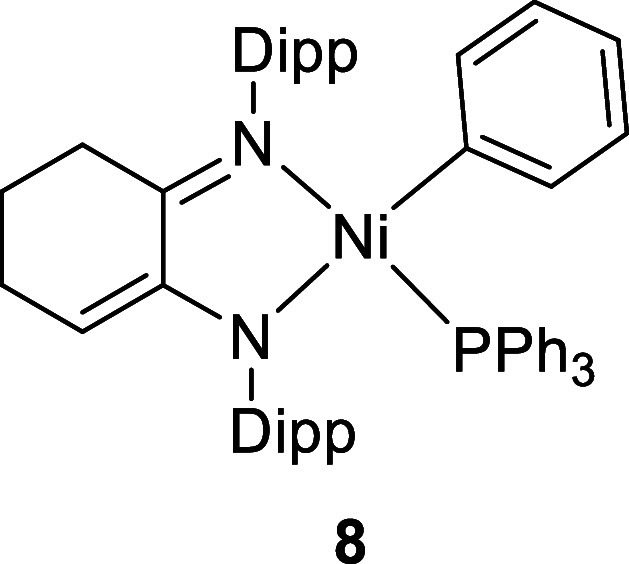


**9** (219 mg, 0.500 mmol 1 equiv) and sodium benzyl (57.0 mg, 0.500 mmol, 1 equiv) were dissolved in benzene (10 mL) and were heated to 50 °C for 1 h. The formed yellow solution was then cooled in an ice bath, allowing the mixture to solidify, and *trans*-[NiCl­(Ph)­(PPh_3_)_2_] was added as a powder. The ice bath was then replaced with a cold-water bath at 7 °C, allowing the solidified solution to melt and mix slowly with the nickel complex. After 1 h, the solvent was evaporated, and the resulting dark brown oil was recrystallized from cold hexane. A second recrystallization from pentane yielded small, plate-shaped dark red crystals (**8**) (124 mg, 30%)

^1^H NMR (400.1 MHz, 298 K, C_6_D_6_) δ/ppm: 7.53 (ddt, *J* = 10.1, 6.7, 1.6 Hz, 6H, ortho-PPh_3_), 7.39 (s, 1H, m-Dipp), 7.35 (s, 1H, m-Dipp), 7.01 (m, 5H, Ar-*H*), 6.87 (m, 11H, overlapping Ar and ortho-Ph), 6.23 (ddd, *J* = 13.9, 7.5, 5.8 Hz, 3H, *m* and *p*-Ph), 4.73 (t, *J* = 5.3 Hz, 1H, C=C*H*), 4.52 (pseudo-hept, 2H, *^i^* Pr-*H*), 3.85 (pseudo-hept, 2H, *^i^* Pr-*H*), 2.16 (t, *J* = 6.3 Hz, 2H, C*H*_*2*_), 1.95 (q, *J* = 5.8 Hz, 2H, C*H*_*2*_), 1.41 (m, *J* = 2H, C*H*_*2*_), 1.37 (d, *J* = 6.8 Hz, 6H, 2 × *^i^* Pr–C*H*_*3*_), 1.22 (d, *J* = 6.8 Hz, 6H 2 × *^i^* Pr–C*H*_*3*_), 1.08 (d, *J* = 6.8 Hz, 6H, 2 × *^i^* Pr–C*H*_*3*_), 0.98 (d, *J* = 6.8 Hz, 6H, 2 × *^i^* Pr–C*H*_*3*_); ^31^P­{^1^H} NMR (162 MHz, 298 K, C_6_D_6_) δ/ppm: 13.90; ^13^C­{^1^H} NMR (100.6 MHz, 298 K, C_6_D_6_) δ/ppm: 155.4 (s, C=*C*–N), 147.2 (s, 4° C), 140.9 (s, 4 °C), 138.36 (d, *J* = 3.0 Hz, 4° C), 136.7 (d, *J*_C–P_ = 4.2 Hz, Ni-Ph), 134.8 (d, *J*_C–P_ = 10.7 Hz, *ortho*-PPh_3_), 134.1 (d, *J*_C–P_ = 13.8 Hz, *ipso*-PPh_3_), 132.3 (d, *J* = 10.1 Hz, Ar CH) 131.8­(s, Ar CH), 129.3 (d, *J*_C–P_ = 1.9 Hz, *para*-PPh_3_), 127.6 (d, *J*_C–P_ = 9.5 Hz, *meta*-PPh_3_), 126.4 (s, Ar CH), 125.1 (narrow doublet, *J*_C–P_ = 1.4 Hz, m-Ph-Ni), 124.1 (s, 4° C), 123.9 (s, Ar CH), 123.7 (s, Ar CH), 121.3 (s, p-Ph-Ni) 89.2 (C), 32.7 (s, CH_2_), 28.9 (s, *^i^* Pr *C*H), 28.8 (s, *^i^* Pr *C*H), 26.8 (s, *^i^* Pr Me), 26.3 (s, CH_2_), 25.0 (s, *^i^* Pr Me), 24.5 (s, CH_2_), 24.1 (s, *^i^* Pr Me), 23.8 (s, *^i^* Pr Me).
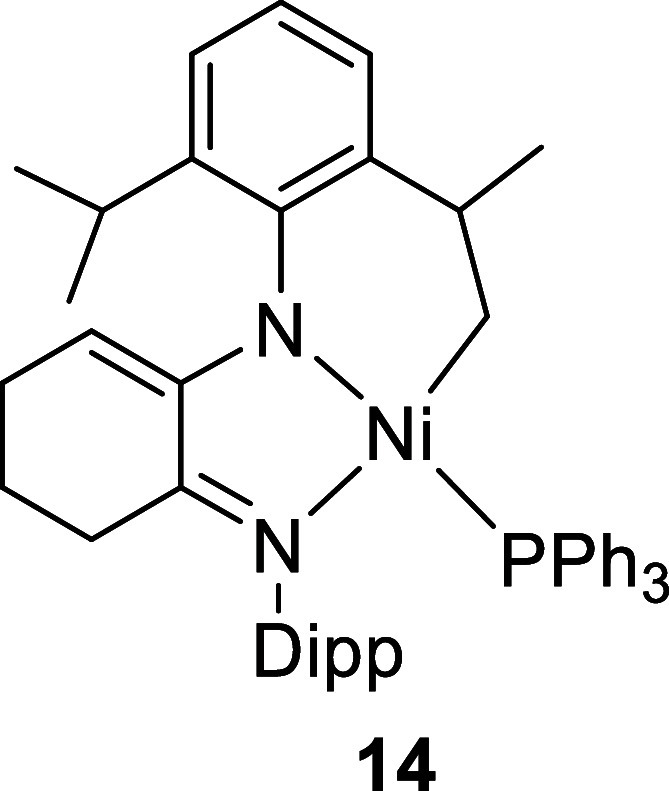


**9** (219 mg, 0.500 mmol 1 equiv) and sodium benzyl (57.0 mg, 0.500 mmol, 1 equiv) were dissolved in benzene (10 mL) and were heated to 50 °C for 1 h. The formed yellow solution was cooled in an ice bath, allowing the mixture to solidify. *trans*-[NiCl­(mesityl)­(PPh_3_)_2_] was added as a powder. The ice bath was then removed, allowing the solidified solution to melt and to mix with the nickel complex slowly at room temperature. After 3 h, the solvent was evaporated, and the resulting dark brown oil was recrystallized from cold hexane to yield dark brown crystals (40 mg, 11%).

^1^H NMR (400.1 MHz, 298 K, C_6_D_6_) δ/ppm: 7.51 (m, 6H, ortho-PPh_3_), 7.43 (dd, *J* = 7.3, 1.9 Hz, 2H, m-Dipp), 6.86 (m, 12H, Ar-*H*), 6.65 (dd, *J* = 7.5, 1.6 Hz, 1H, Ar-*H*), 5.19 (t, *J* = 5.2 Hz, 1H, Ar*H*), 4.30 (hept, *J* = 6.8 Hz, 1H, *^i^* Pr-*H*), 3.76 (pseudo-hept, *J* = 6.8 Hz, 1H, *^i^* Pr-*H*), 3.10 (2H; coincident pseudo-hept, *J* = 13.6, 6.8 Hz, *^i^* Pr-*H* and CH), 2.07 (m, 2H, C*H*_*2*_), 1.86 (ddt, *J* = 20.6, 9.9, 5.0 Hz, 2H, C*H*_*2*_), 1.74 (d, *J* = 6.8 Hz, 3H, *^i^* Pr–C*H*_*3*_), 1.55 (d, *J* = 6.9 Hz, 3H, *^i^* Pr–C*H*_*3*_), 1.44 (m, 3H, overlapping C*H*_*2*_ and C*H*_a_ of bridging CH_2_), 1.20 (dd, *J* = 6.8, 3.8 Hz, 6H, *^i^* Pr–C*H*_*3*_), 1.16 (d, *J* = 6.8 Hz, 3H, *^i^* Pr–C*H*_*3*_), 1.03 (d, *J* = 6.8 Hz, 3H, *^i^* Pr–C*H*_*3*_), 0.84 (d, *J* = 6.8 Hz, 3H, *^i^* Pr–C*H*_*3*_), 0.10 (ddd, *J* = 11.5, 9.7, 6.1 Hz, 1H, C*H*_b_); ^13^C­{^1^H}­NMR (100.6 MHz, 298 K, C_6_D_6_) δ/ppm: 177.52 (d, *J*_C–P_ = 3.2 Hz, *C*=N), 151.18 (s, C=*C*–N), 146.6 (s, Ar C), 146.2 (s, Ar C), 143.9 (s, Ar C), 141.1 (d, *J*_C–P_ = 1.8 Hz, Ar C), 139.4 (d, *J*_C–P_ = 2.2 Hz, 4° Ar C), 134.5 (d, *J*_C–P_ = 11.2 Hz, *ipso*-PPh_3_), 133.9 (s, Ar C), 133.6 (s, 4° Ar C), 129.1 (d, *J*_C–P_ = 1.8 Hz *para*-PPh_3_), 128.7 (s, 4° Ar C), 126.1 (s, Ar CH), 123.9 (s, Ar CH), 123.6 (s, Ar CH), 123.0 (s, Ar CH), 121.6 (s, Ar CH), 121.0 (s, Ar CH), 106.11 (d, *J*_C–P_ = 2.3 Hz, HC = CH_2_), 35.8 (s, CH_2_), 35.40 (d, *J*_C–P_ = 3.5 Hz, *C*H), 31.96 (s, C), 31.46 (s, CH_2_), 29.0 (s, *^i^* Pr *C*H), 28.8 (s, *^i^* Pr *C*H), 28.4 (s, *^i^* Pr *C*H), 26.43 (s, *^i^* Pr Me), 25.99 (s, CH_2_), 24.68 (s, *^i^* Pr Me), 24.2 (s, CH_2_), 24.1 (s, *^i^* Pr Me), 24.0 (s, *^i^* Pr Me), 22.8 (s, *^i^* Pr Me), 22.2 (s, *^i^* Pr Me), 19.57 (d, *J*_C–P_ = 2.8 Hz, CH_3_); ^31^P­{^1^H} NMR (162 MHz, 298 K, C_6_D_6_) δ/ppm: 30.3.

### Polymerization Details

Polymerization reactions were run using a magnetically stirred Parr 9010 0.45 L autoclave. A typical polymerization reaction is as follows: In the glovebox, a Schlenk flask was charged with 10.0 mg of the Ni precatalyst (0.0120 mmol) and B­(C_6_F_5_)_3_ (24.7 mg, 0.0483 mmol, 4.0 equiv). This was then transferred to a Schlenk line under N_2_, and toluene (30 mL) was added. This homogeneous solution was quickly transferred via a cannula to a dry and preheated (or cooled) Parr autoclave. The autoclave was then rapidly pressurized with ethylene, and the polymerization was carried out under a fixed pressure. After 30 min (or 60 min for the 0 °C reaction), the reactor release valve was opened. A small sample of the reaction solution was then taken, and the ^1^H NMR spectrum was run to ascertain if any oligomers or polymers were retained in solution (nothing was observed). Acetone was then added to the solution to complete the precipitation of the polymer. This polymer was then filtered and dried before weighing and subsequent analysis.

### Polymer Analysis

PE samples were analyzed as 1,2,4-trichlorobenzene solutions (15 mg in 10 mL) at 160 °C using an Agilent Technologies PL GPC220 with an Agilent Technologies PLgel Olexis guard plus 3 × Olexis, 30 cm, 13 μm column, featuring a refractive index detector with differential pressure and light scattering. The HT-GPC system used for this work was calibrated using a series of Agilent/Polymer Laboratories EasiVial PS-H polystyrene calibrants with known molecular weights; however, a mathematical correction, making use of Mark–Houwink parameters, had been applied to express the results as for the linear PE homopolymer (see Table S3 for the Mark–Houwink parameters used).

## Supplementary Material



## Notes

Experimental details, full characterization data, and crystallographic details for the complexes described (PDF)

## †

School of Chemistry, Joseph Black Building, University of Glasgow, Glasgow G12 8QQ, U.K
