# Supplementary material for: Design of Neutral Ni[N,N] Catalysts for High-Density Polyethylene Formation: Insights into Catalyst Deactivation
Source: Organometallics. 2025 Sep 26;44(19):2163–71. doi: 10.1021/acs.organomet.5c00186 (PMC12522148; doi:10.1021/acs.organomet.5c00186)
Supplement: Supplementary file 1 [file om5c00186_si_001.pdf]

Supporting Information

The Design of Neutral Ni[N,N] Catalysts for High-Density Polyethylene Formation: Insights into Catalyst Deactivation

Bence Szabó, Lennox W. Stewart, Georgina M. Rosair and Stephen M. Mansell*

Institute of Chemical Sciences, Heriot-Watt University, Edinburgh, EH14 4AS, UK.

E-mail: s.mansell@hw.ac.uk, https://www.mansellresearch.org.uk

1 Contents

2 NMR spectra and additional experimental details 2
2.1 NMR spectra of **12** 2
2.2 NMR spectra for **10** 3
2.3 NMR spectra for **13** 4
2.4 NMR spectra for **7** 5
2.5 NMR spectra of **8** 8
2.6 NMR spectra of **14** 10
2.7 Decomposition of **7** 13
2.8 Decomposition of **8** 15
3 X-ray crystallography 17
3.1 Crystallographic details 17
3.2 Additional structures 17
3.2.1 **10** 17
3.2.2 Additional perspective of **14** 18
3.3 Crystallographic tables of data 19
4 Polymer characterisation 21
4.1 DSC 21
4.2 High temperature GPC 25
5 References 26

2 NMR spectra and additional experimental details

2.1 NMR spectra of 12

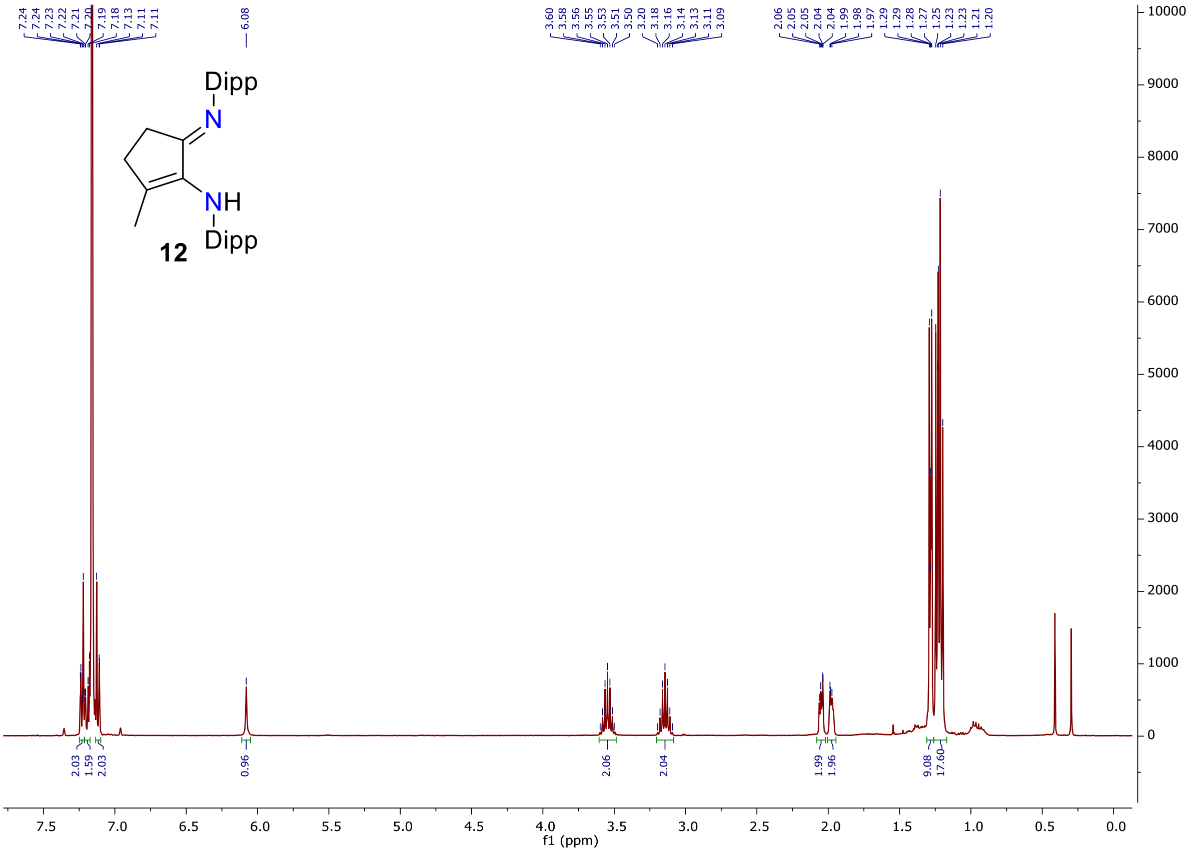

Figure S1. ^{1}H NMR spectrum (400 MHz, C_6D_6, 298 K) of **12**.

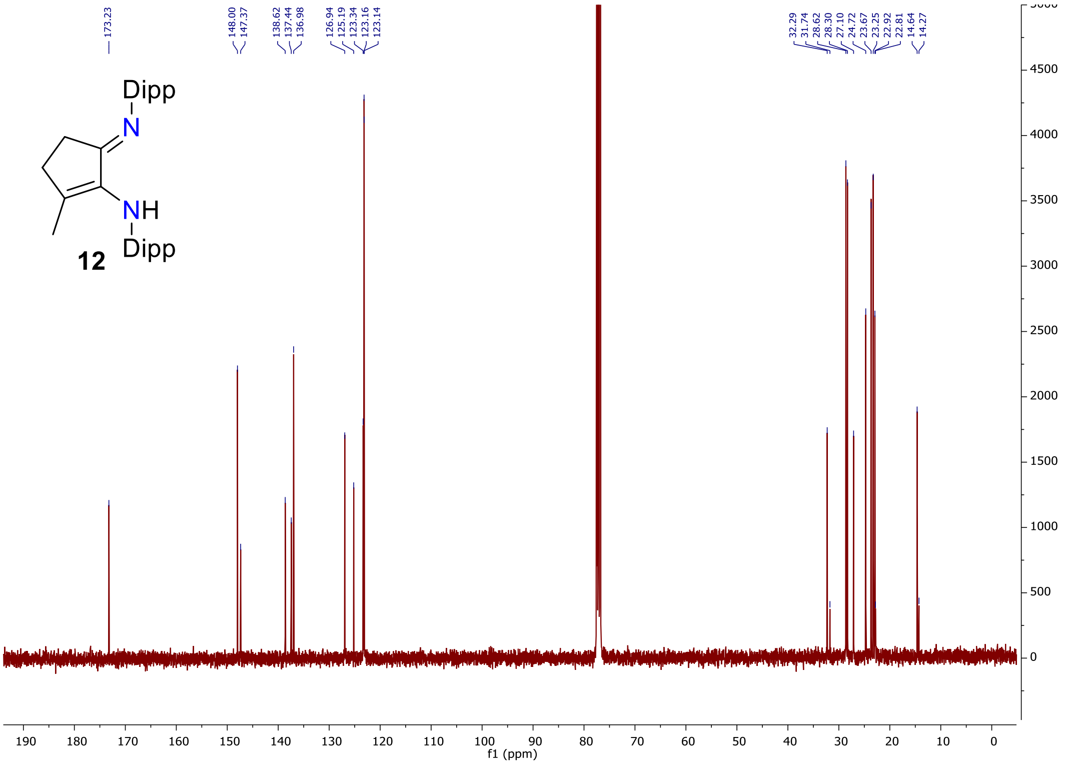

Figure S2. $^{13}C\{^{1}H\}$ NMR spectrum (101 MHz, C_6D_6, 298 K) of **12**.

2.2 NMR spectra for 10

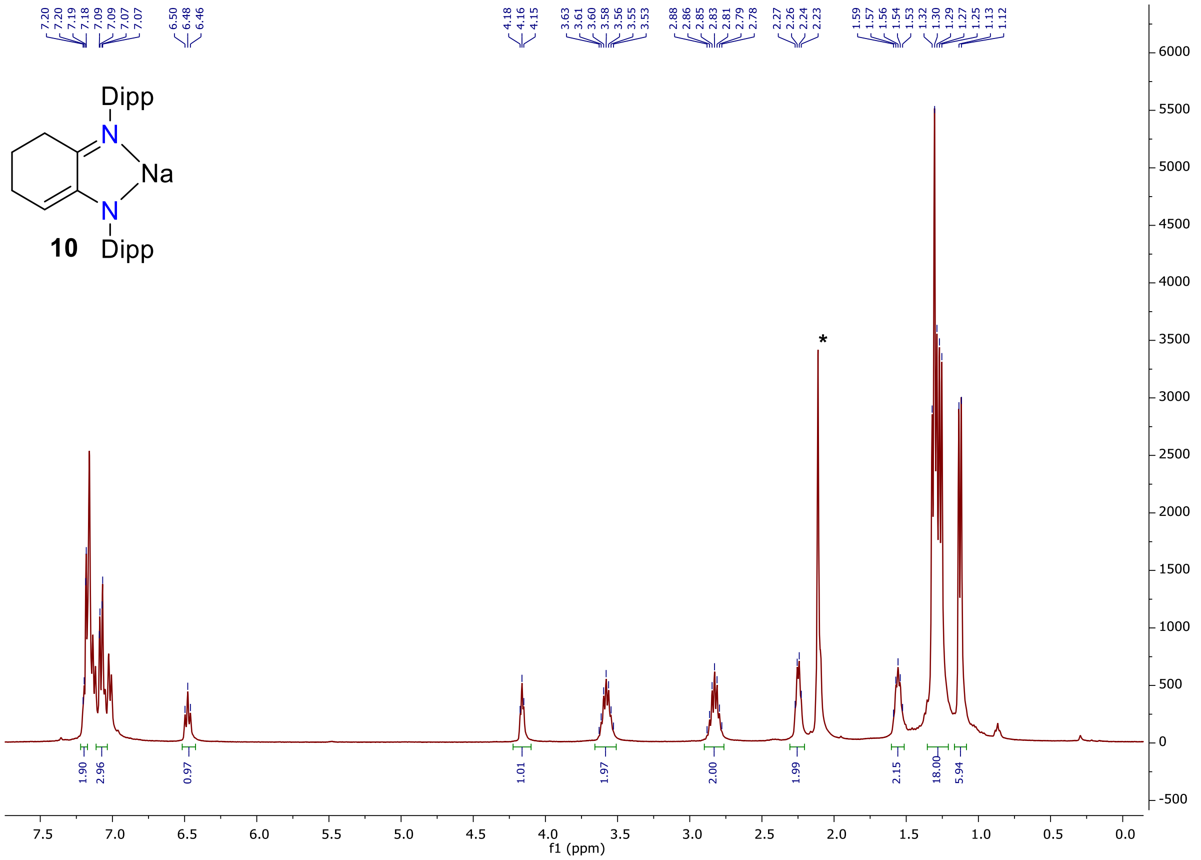

Figure S3. ^{1}H NMR spectrum (400 MHz, C_6D_6, 298 K) of **10**. * indicates one equivalent of toluene.

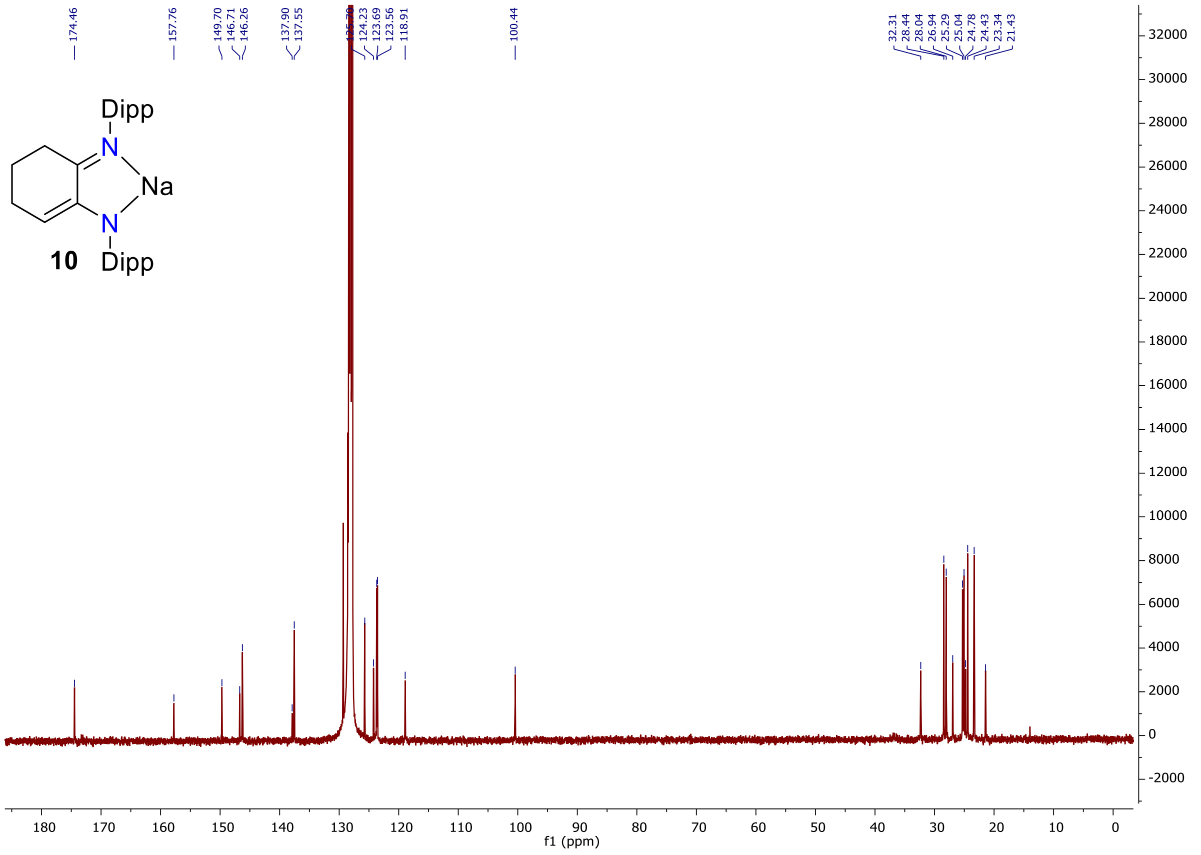

Figure S4. $^{13}C\{^{1}H\}$ NMR spectrum (101 MHz, C_6D_6, 298 K) of **10**.

2.3 NMR spectra for 13

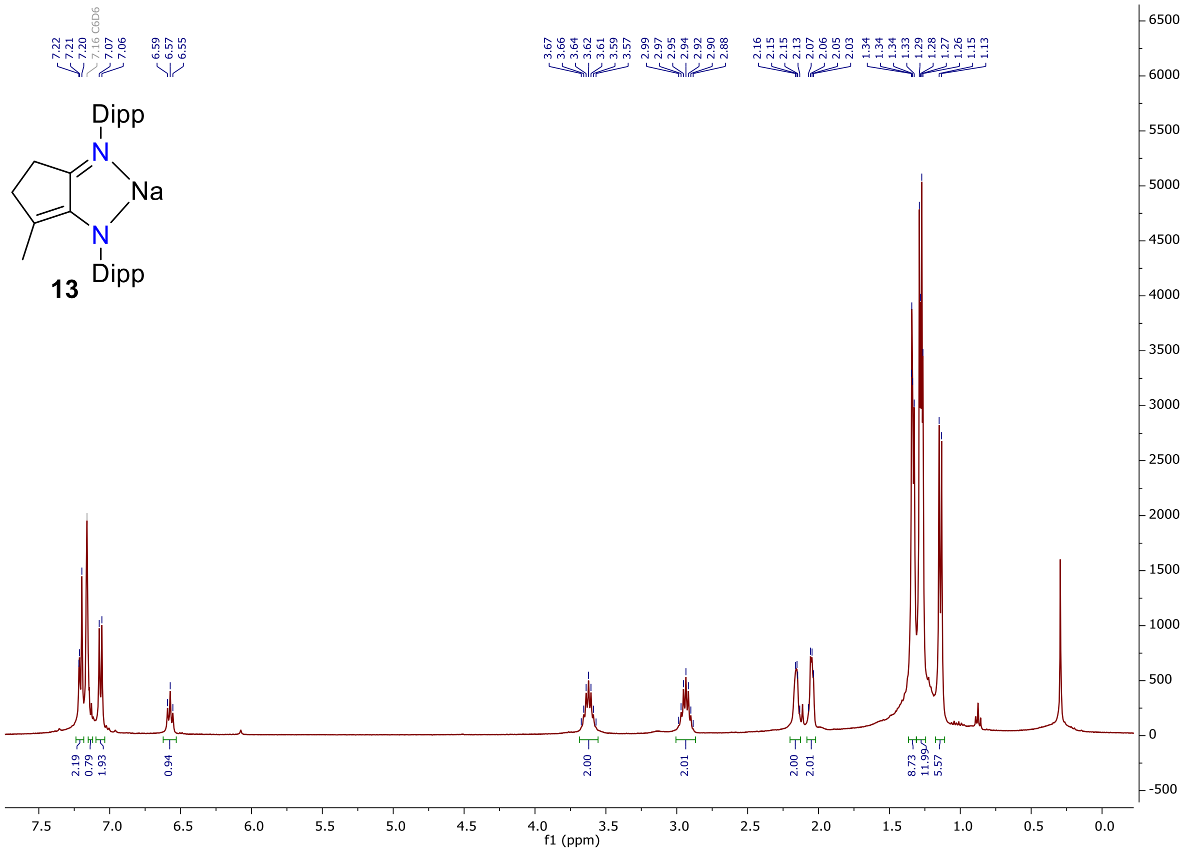

Figure S5. ^{1}H NMR spectrum (400 MHz, C_6D_6, 298 K) of **13**. Toluene formed in the reaction is also present.

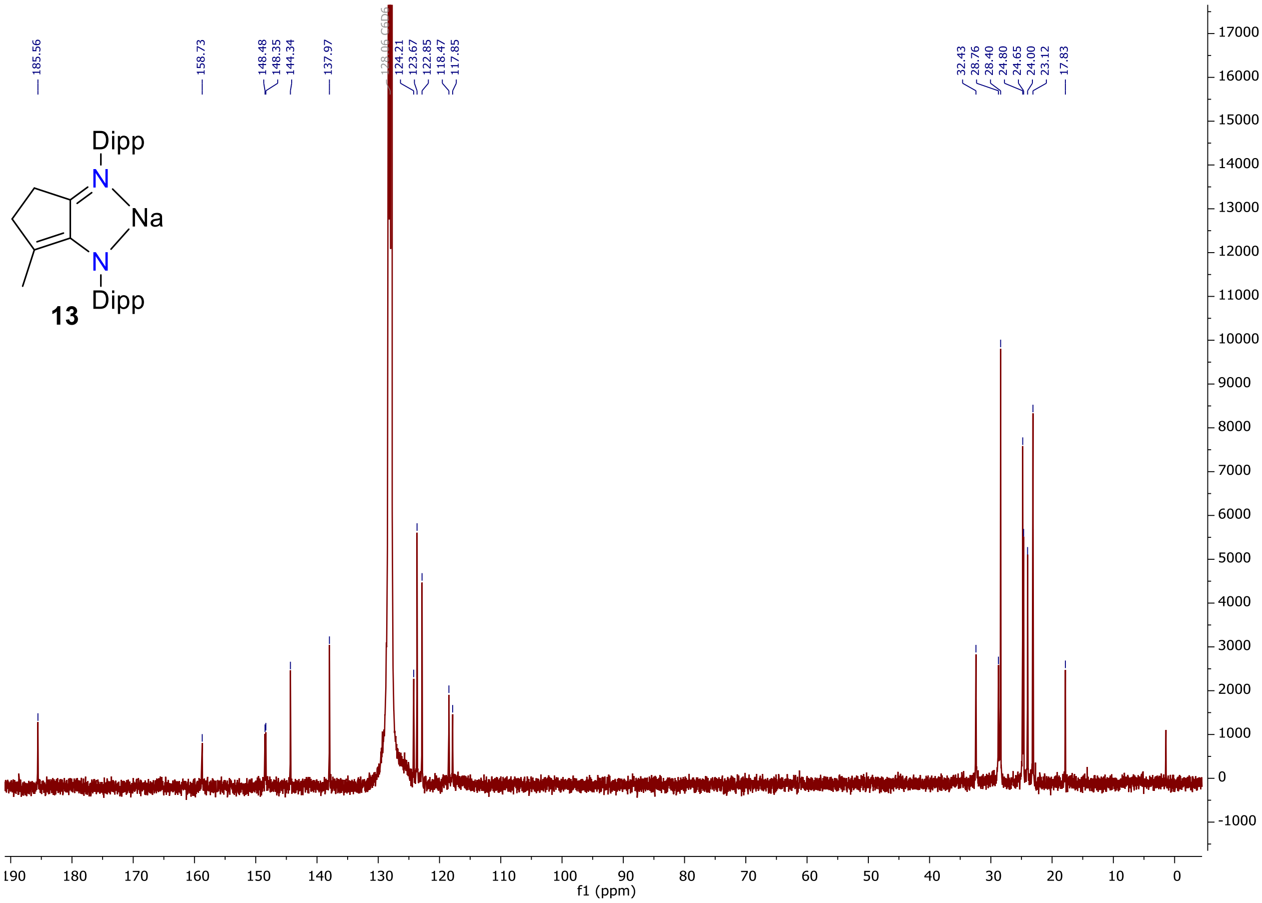

Figure S6. $^{13}C\{^{1}H\}$ NMR spectrum (101 MHz, C_6D_6, 298 K) of **13**.

2.4 NMR spectra for 7

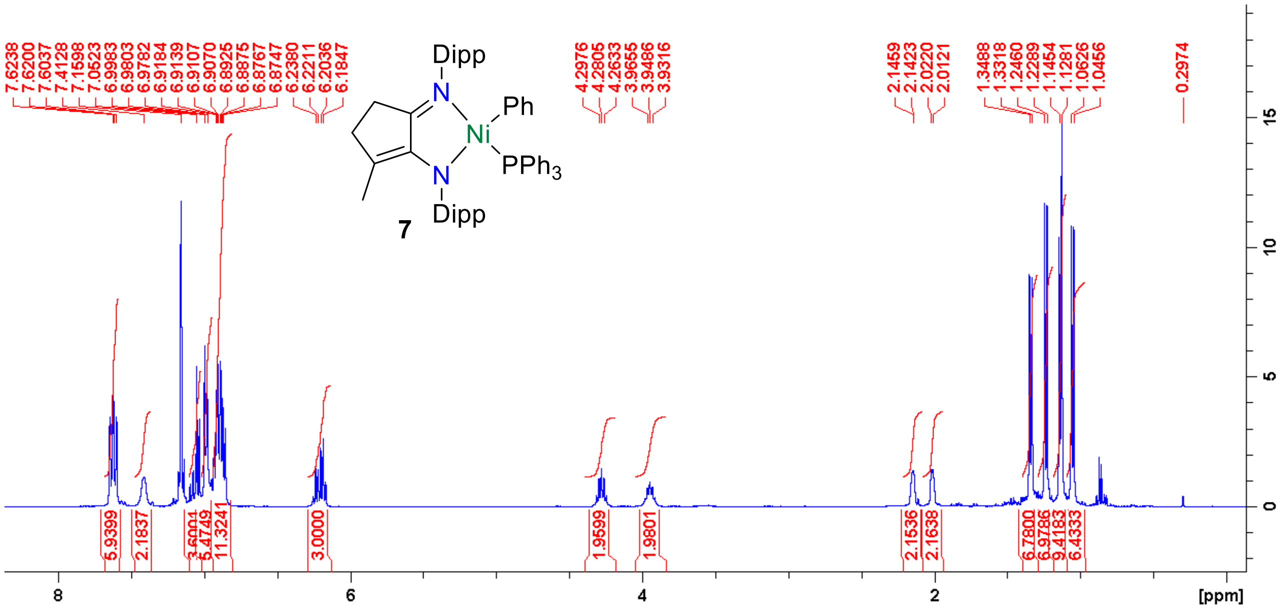

Figure S7. ^{1}H NMR spectrum (400 MHz, C_6D_6, 298 K) of **7**.

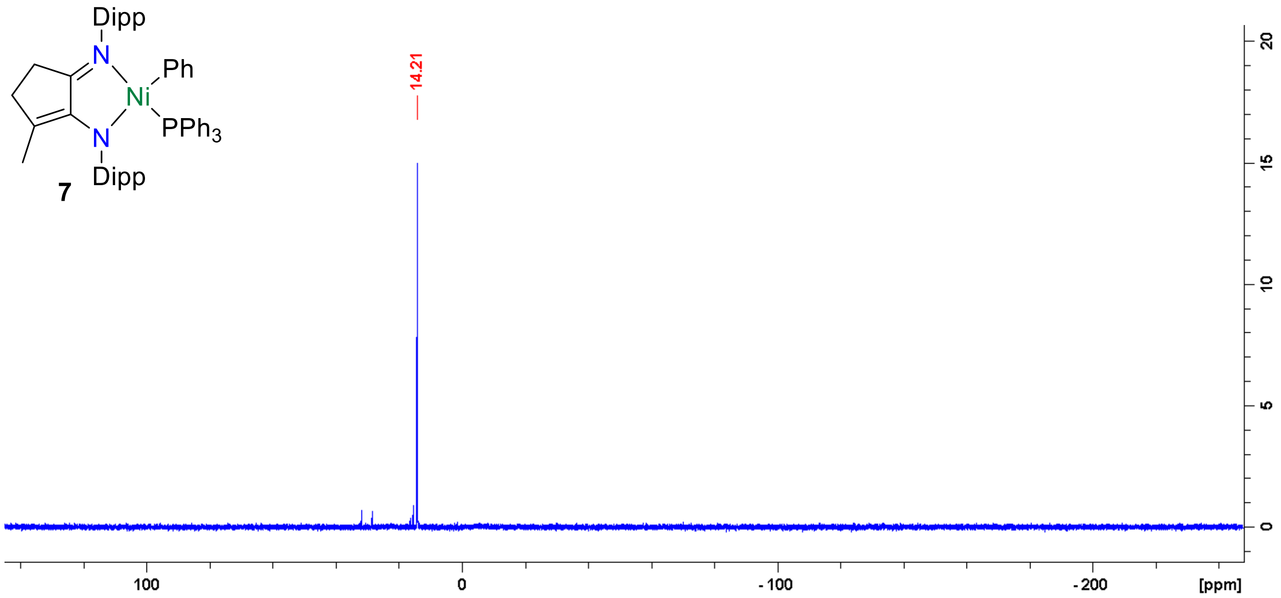

Figure S8. $^{31}P\{^{1}H\}$ NMR spectrum (162 MHz, C_6D_6, 298 K) of **7**.

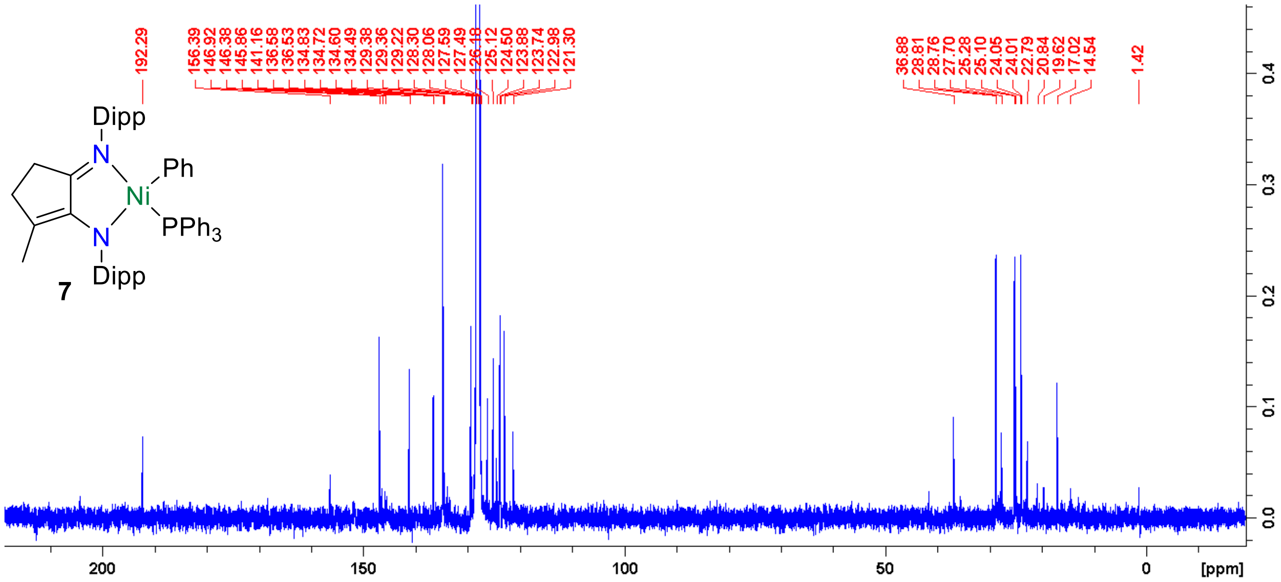

Figure S9. $^{13}C\{^{1}H\}$ NMR spectrum (101 MHz, C_6D_6, 298 K) of **7**.

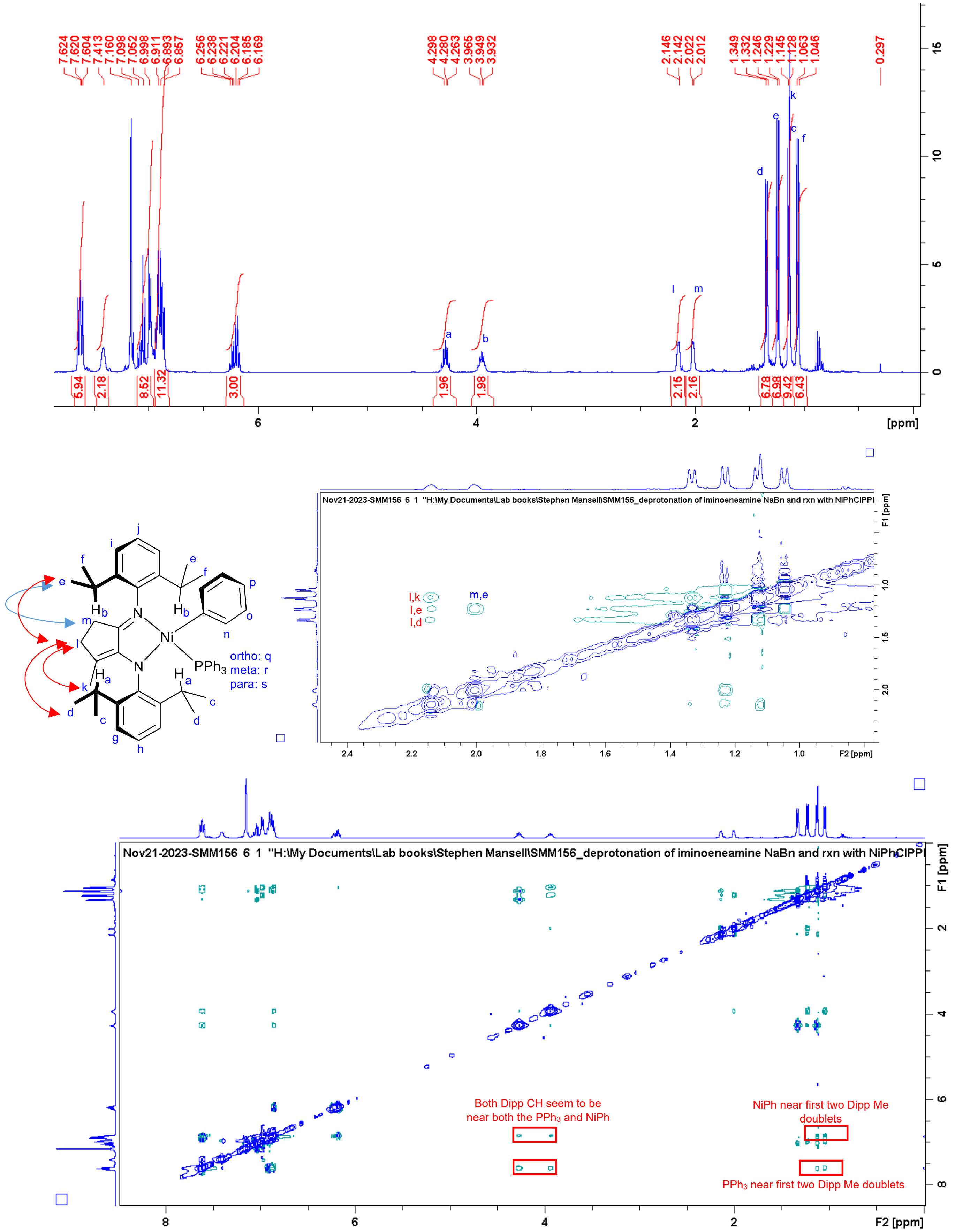

Figure S10. Additional assignment of **7** was aided by 2D NMR spectroscopy (COSY, HSQC, HMBS and NOESY; only NOESY spectra are shown).

2.5 NMR spectra of 8

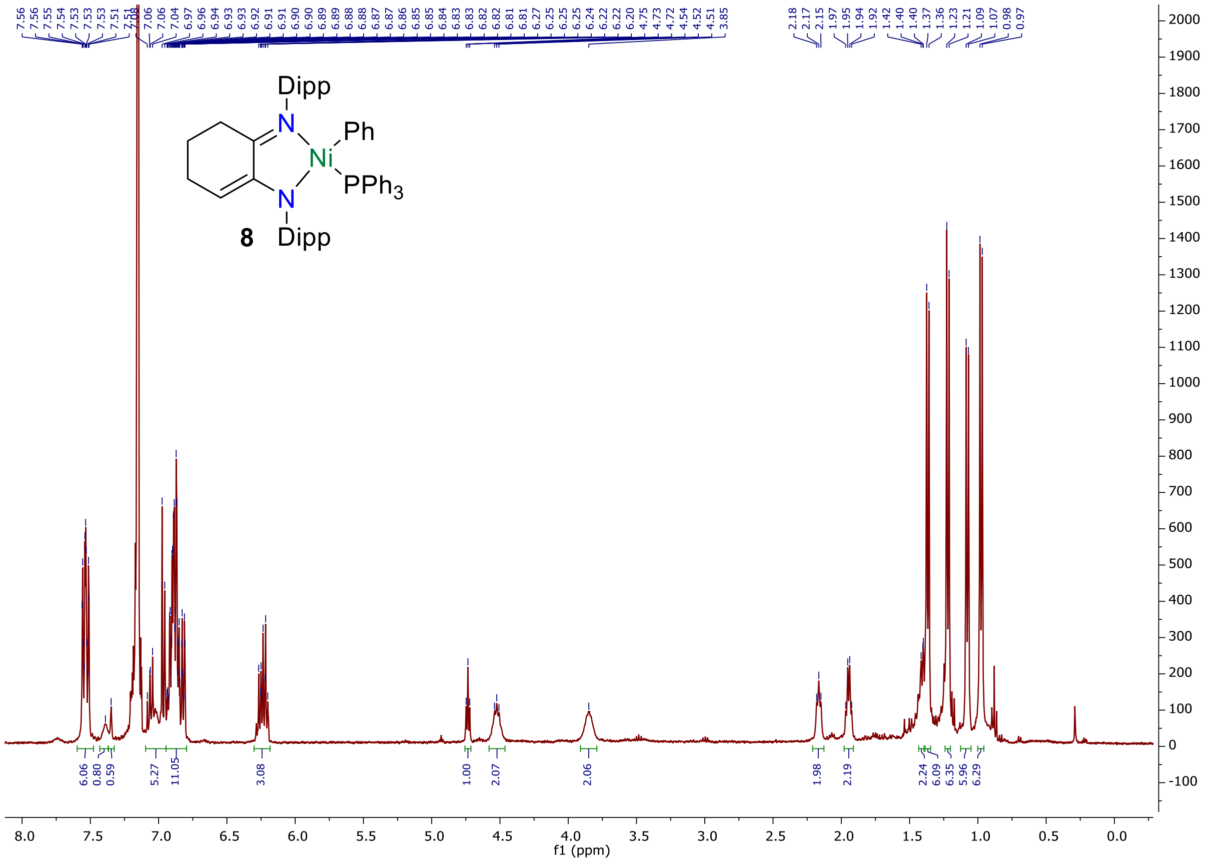

Figure S11. ^{1}H NMR spectrum (400 MHz, C_6D_6, 298 K) of **8**.

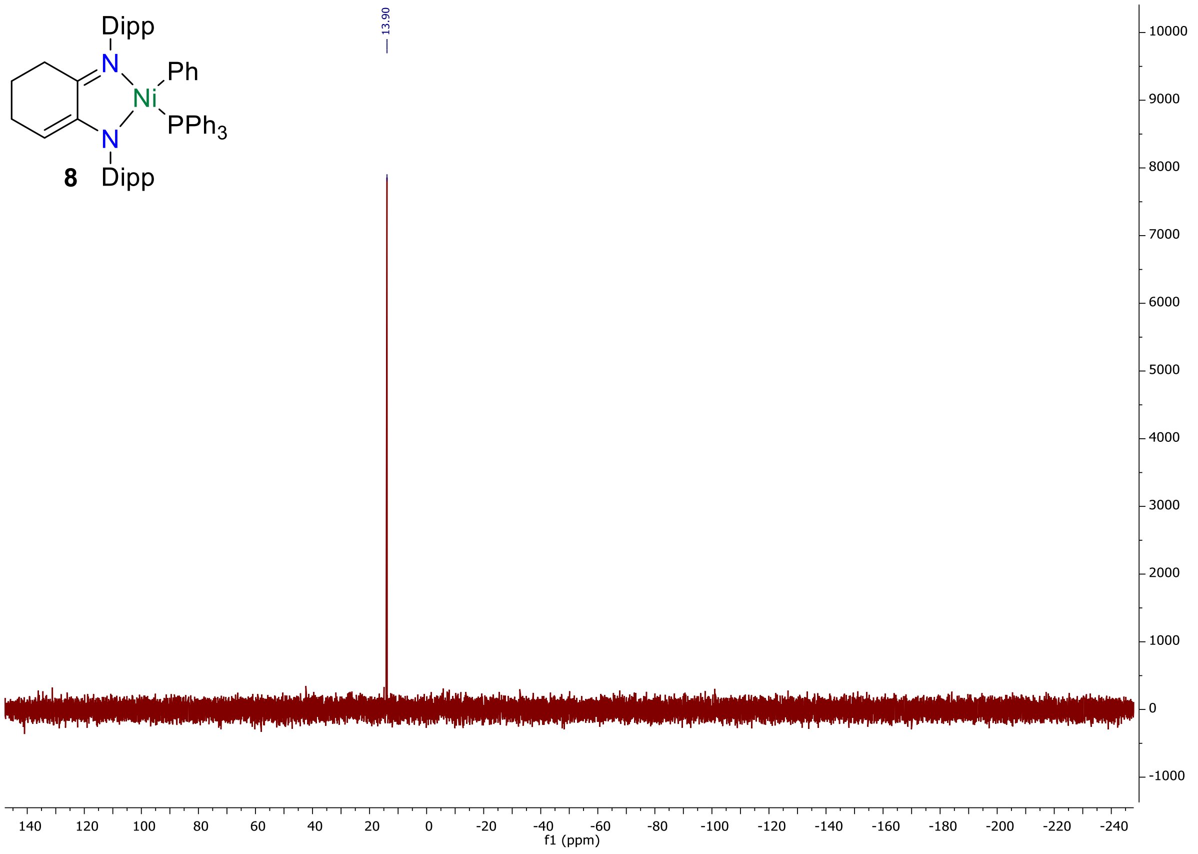

Figure S12. $^{31}P\{^{1}H\}$ NMR spectrum (162 MHz, C_6D_6, 298 K) of **8**.

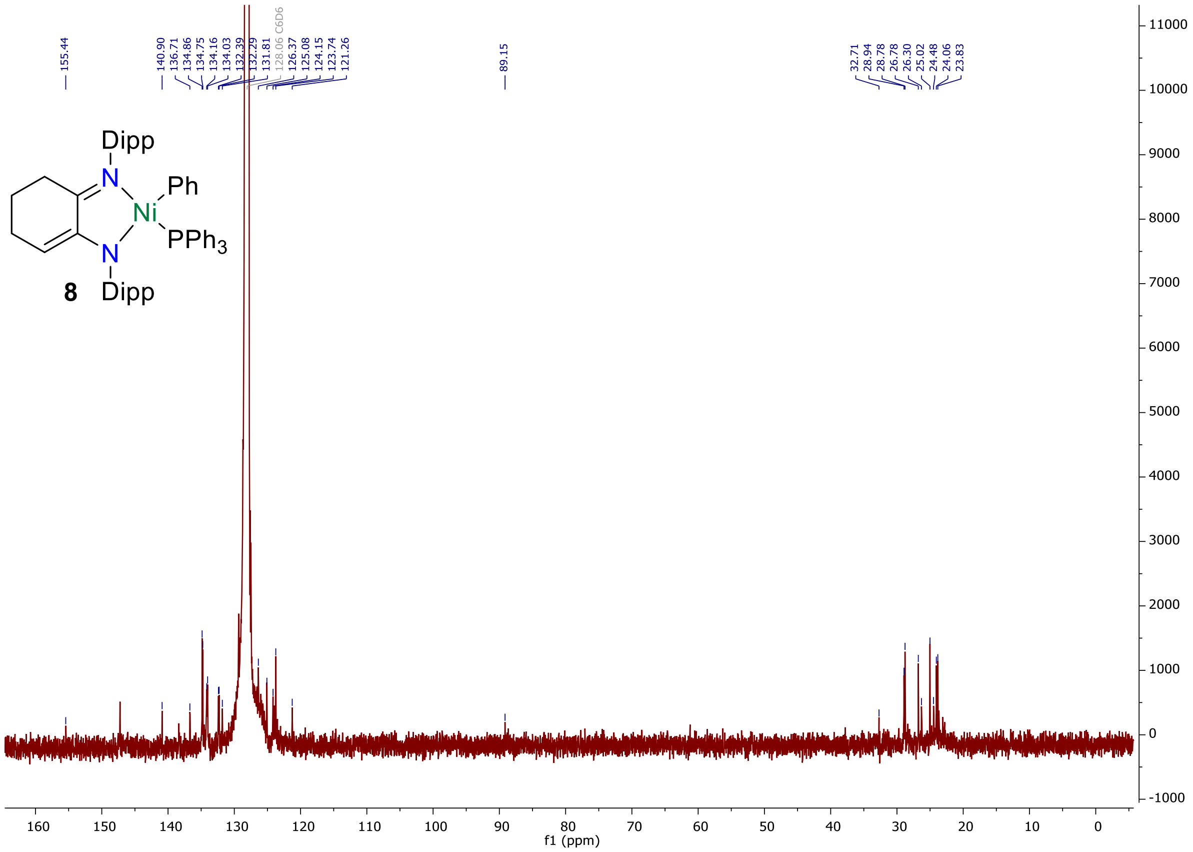

Figure S13. $^{13}C\{^{1}H\}$ NMR spectrum (101 MHz, C_6D_6, 298 K) of **8**.

2.6 NMR spectra of 14

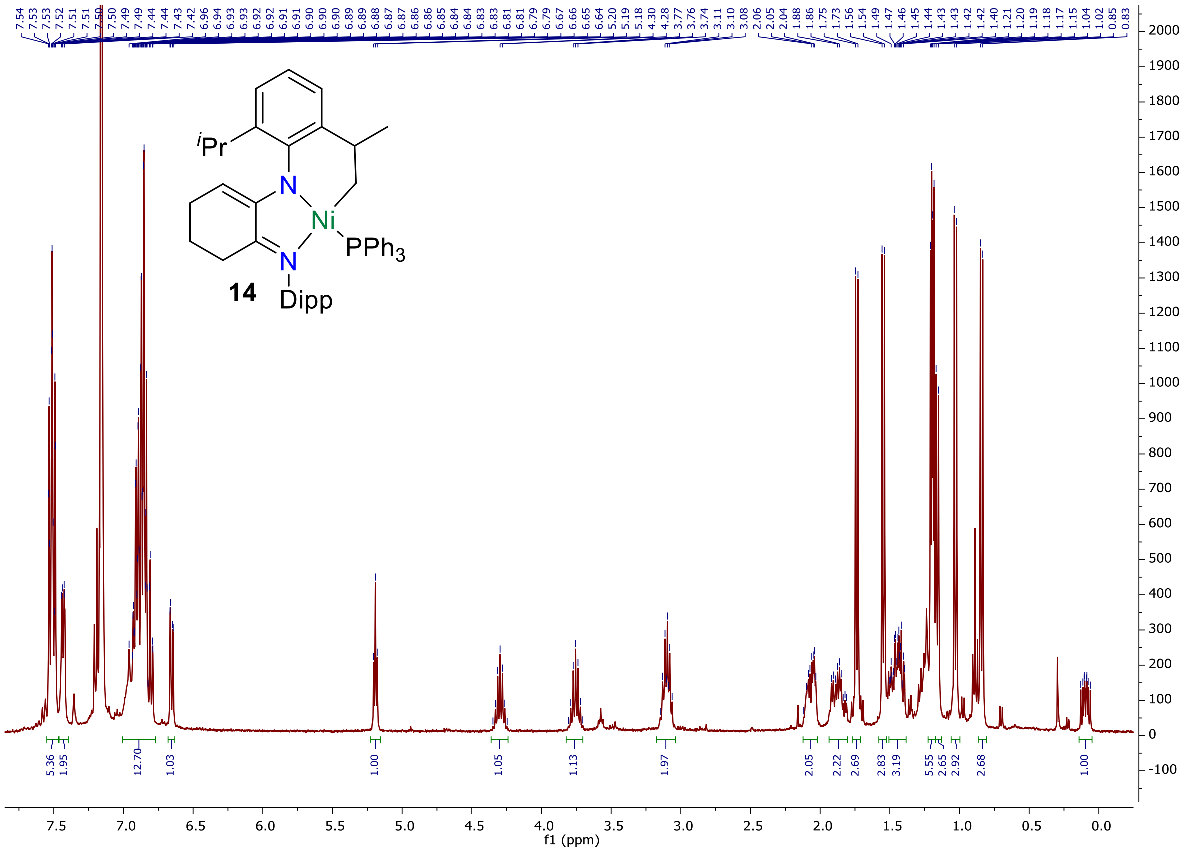

Figure S14. ^{1}H NMR spectrum (400 MHz, C_6D_6, 298 K) of **14**.

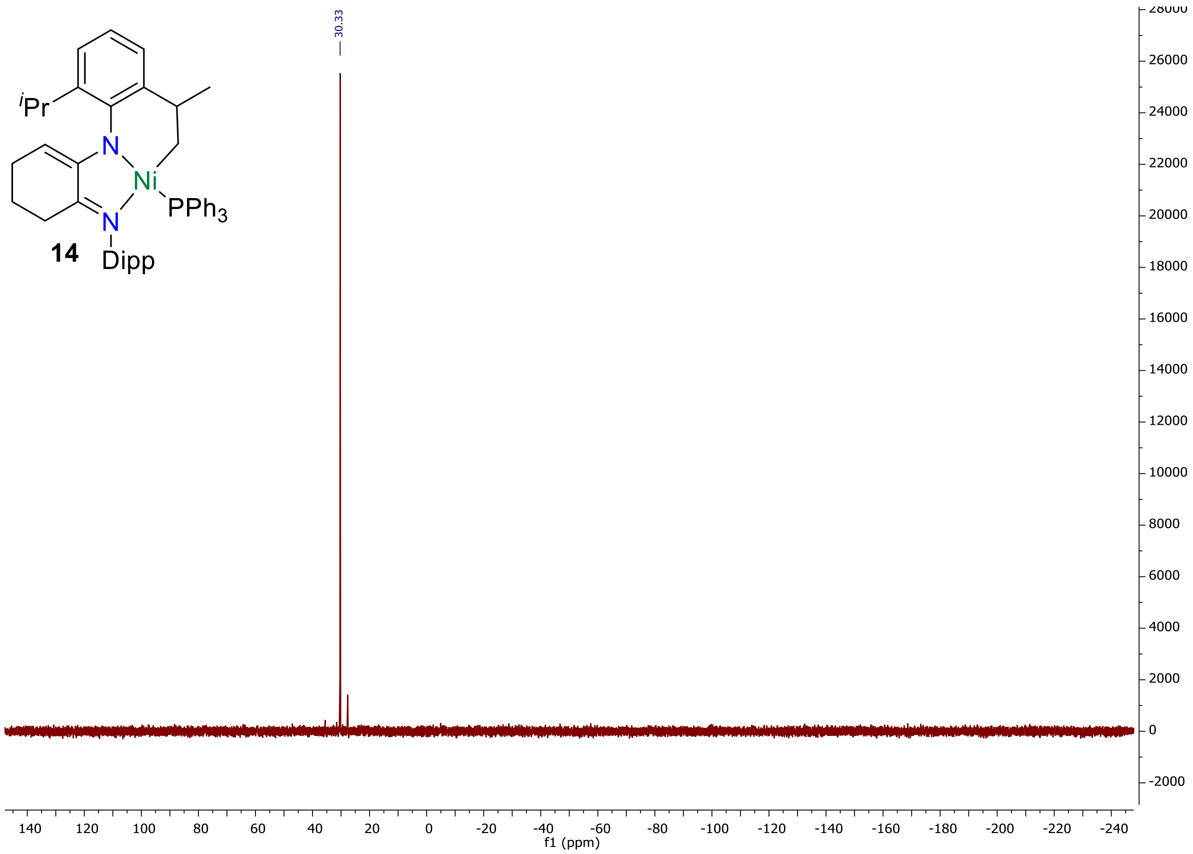

Figure S15. $^{31}P\{^{1}H\}$ NMR spectrum (162 MHz, C_6D_6, 298 K) of **14**.

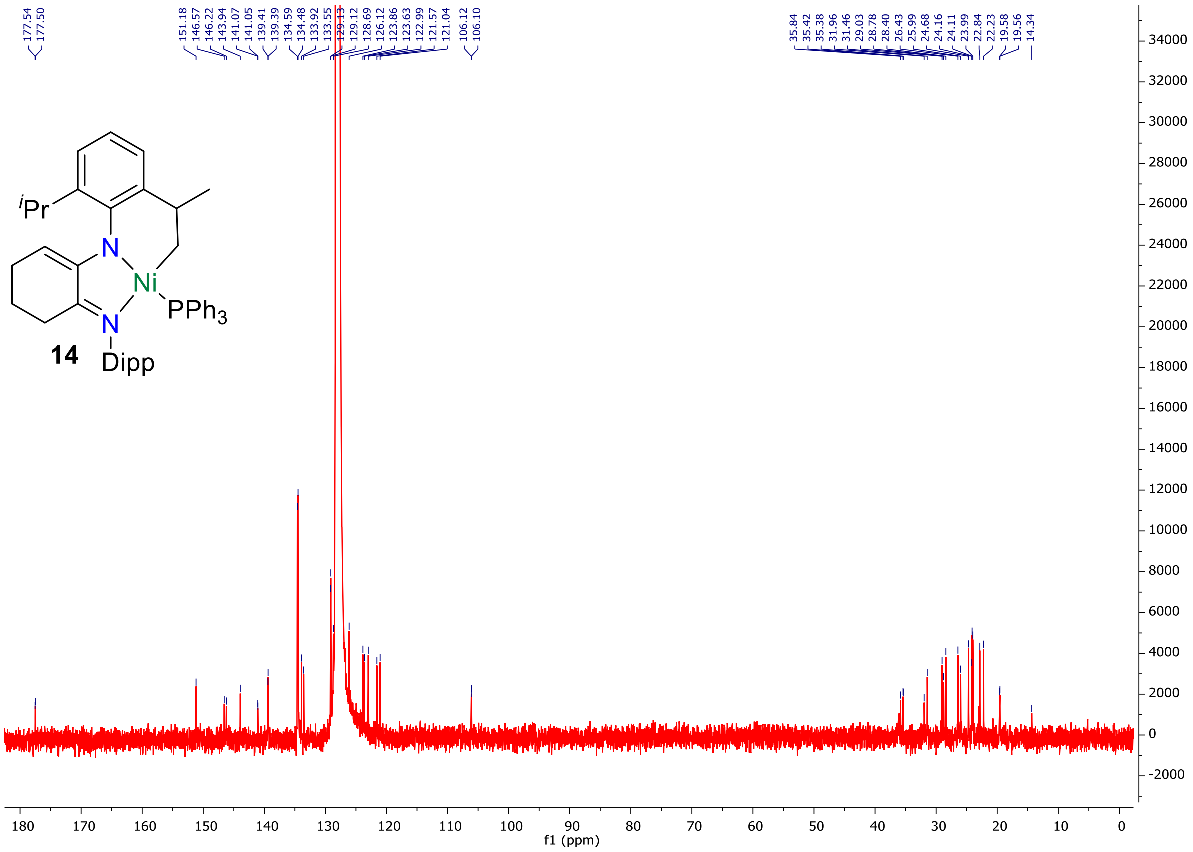

Figure S16. $^{13}C\{^{1}H\}$ NMR spectrum (101 MHz, C_6D_6, 298 K) of **14**.

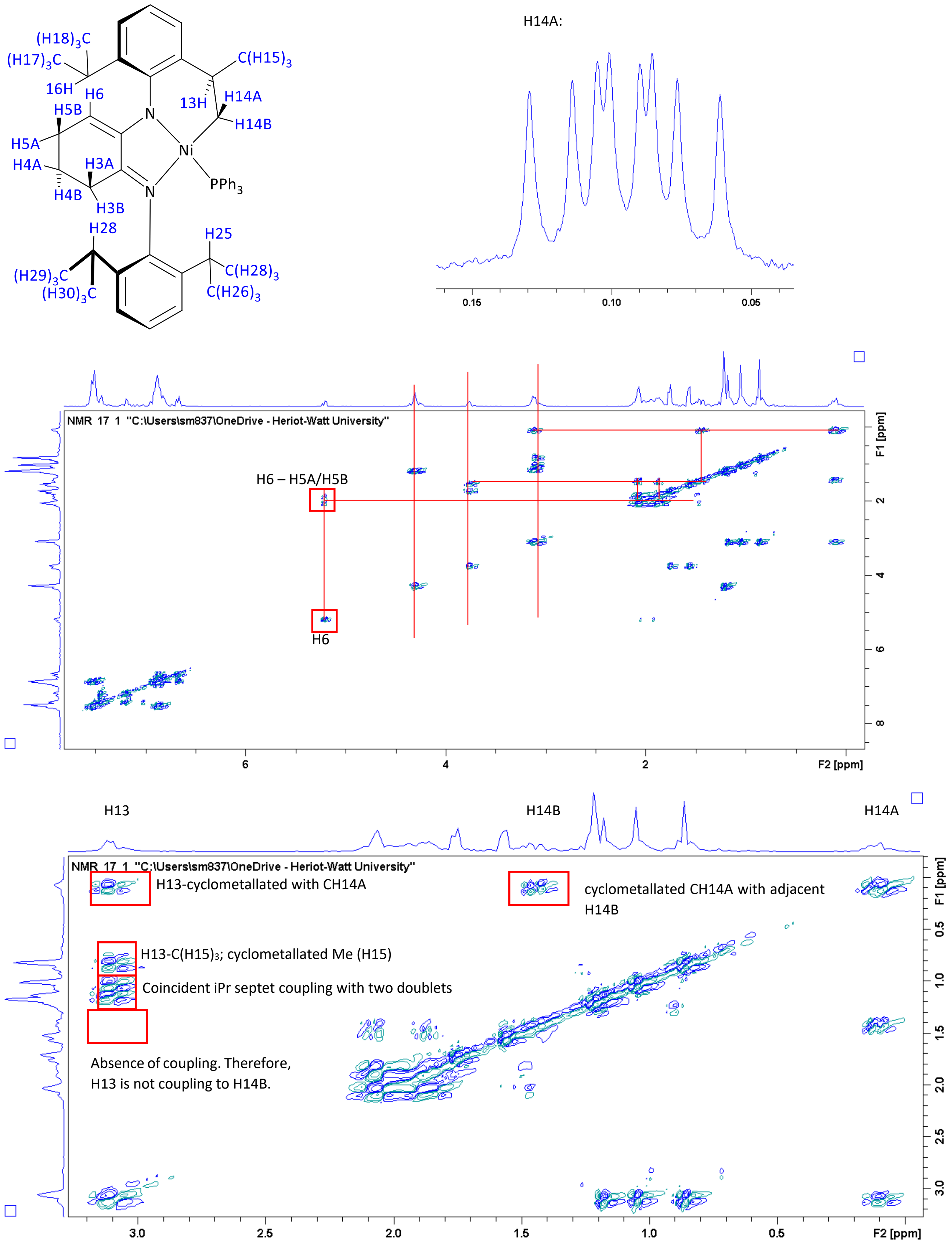

Figure S17. COSY spectrum for **14** (top) and zoomed in region (bottom). A red box shows the absence of coupling which helped assign H14B (Karplus equation).

2.7 Decomposition of 7

A C_6D_6 solution of **7** was left at room temperature for 16 days or heated at 80 °C overnight.

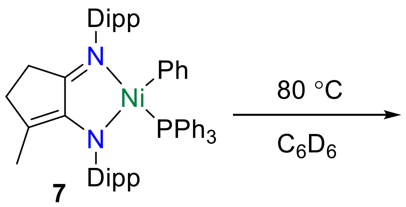

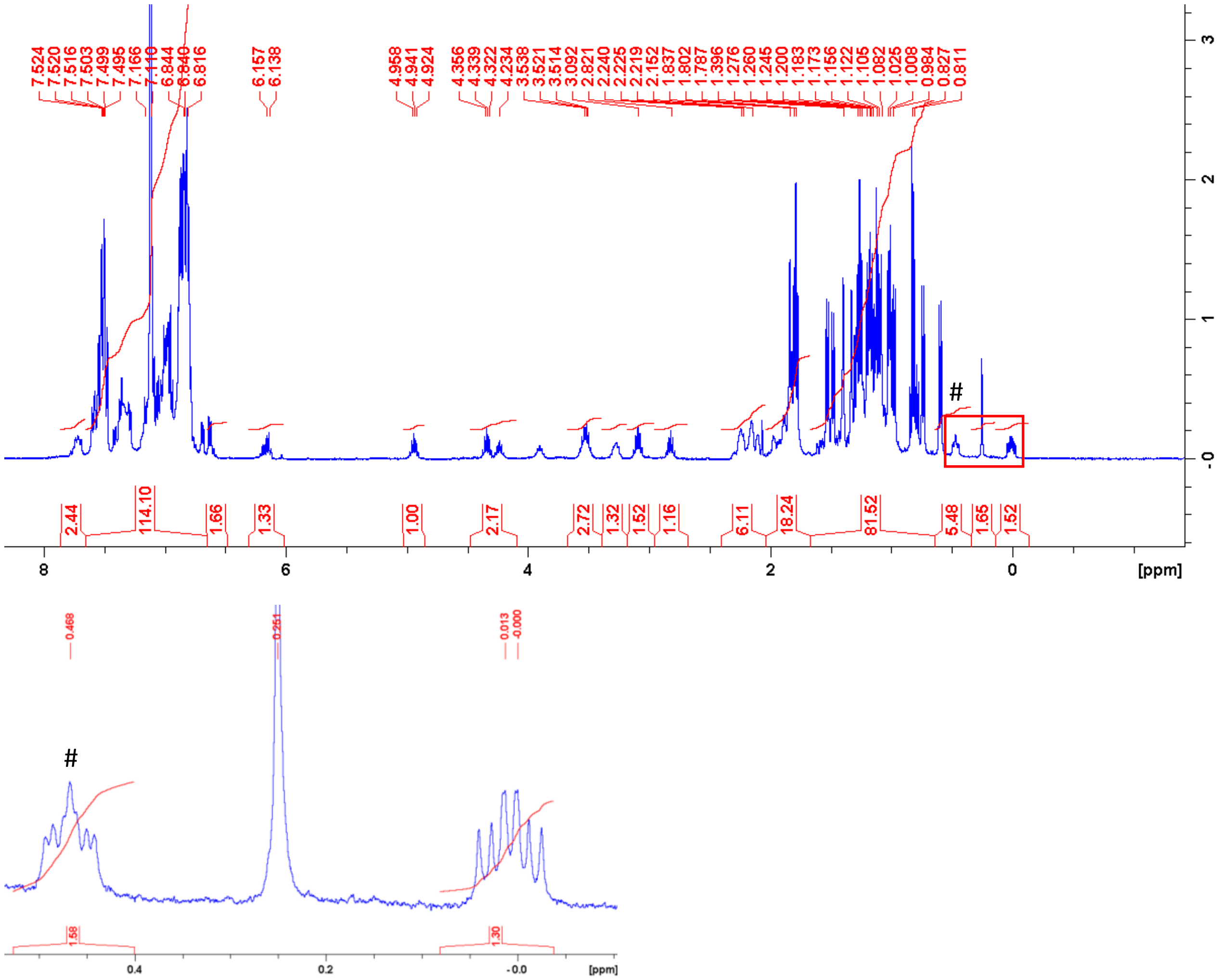

Figure S18. ^{1}H NMR spectrum (400 MHz, C_6D_6, 298 K) from the thermal decomposition of **7** (top); zoomed in region around 0 ppm (bottom) reveals two characteristic multiplets. The likely presence of a second, unidentified product is indicated by the characteristic multiplet labelled with #.

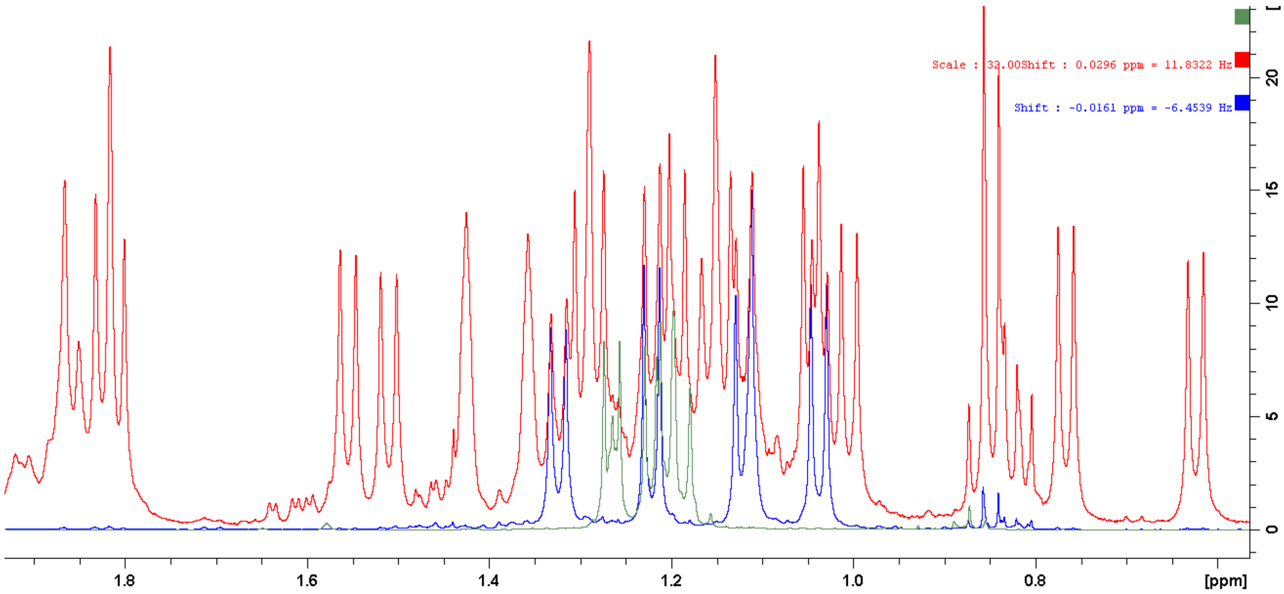

Figure S19. Zoomed in region of the ^{1}H NMR spectrum (400 MHz, C_6D_6, 298 K) from the thermal decomposition of **7** (red). This region shows the methyl groups. This spectrum (red) is contrasted with the protonated ligand (**12**, green) and the starting complex (blue, **7**).

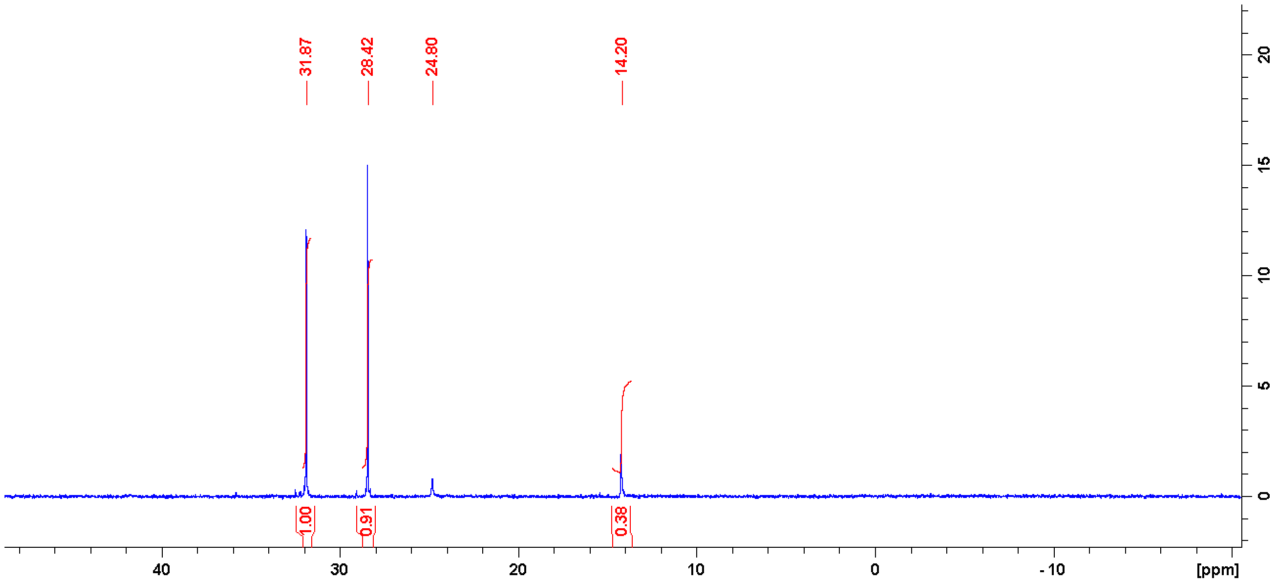

Figure S20. $^{31}P\{^1H\}$ NMR spectrum (162 MHz, C_6D_6, 298 K) from the thermal decomposition of **7**. The singlet at 14.2 ppm is from residual **7**.

2.8 Decomposition of 8

A C_6D_6 solution of **8** was left at room temperature for 18 h.

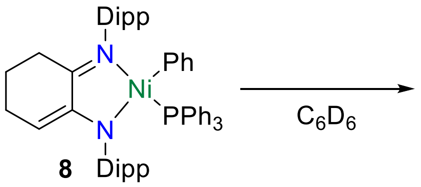

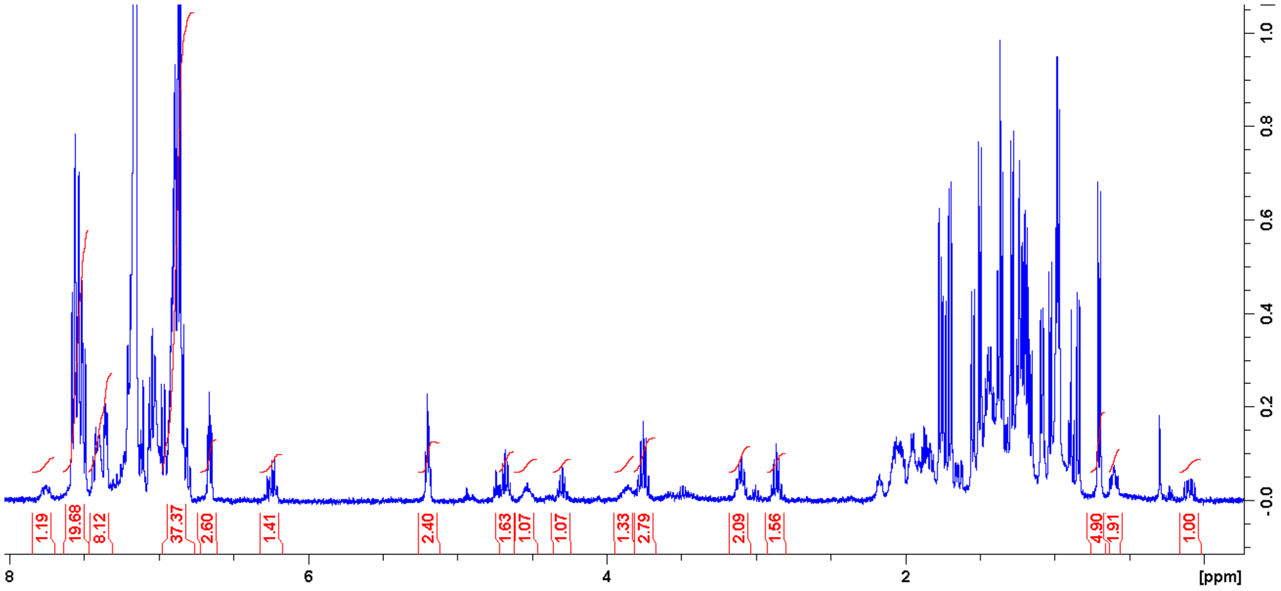

Figure S21. ^{1}H NMR spectrum (400 MHz, C_6D_6, 298 K) from the thermal decomposition of **8**.

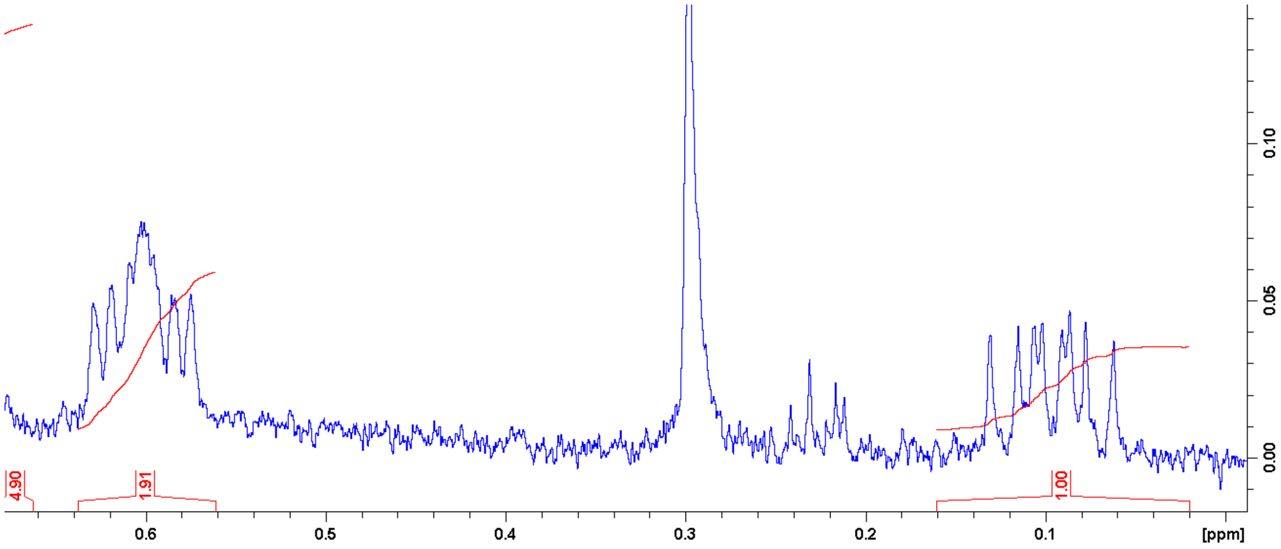

Figure S22. Zoomed in region of the ^{1}H NMR spectrum of the thermal decomposition of **8**.

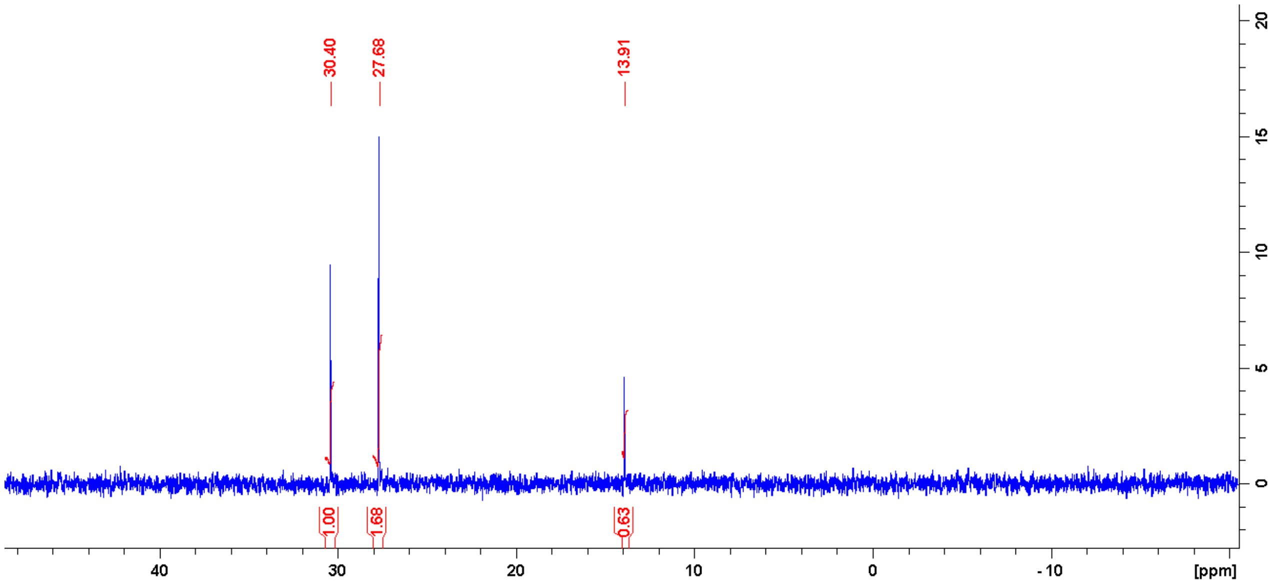

Figure S23. $^{31}P\{^1H\}$ NMR spectrum (162 MHz, C_6D_6, 298 K) from the thermal decomposition of **8**. The singlet at 30.4 ppm is from **14**; the singlet at 13.9 ppm is from **8**.

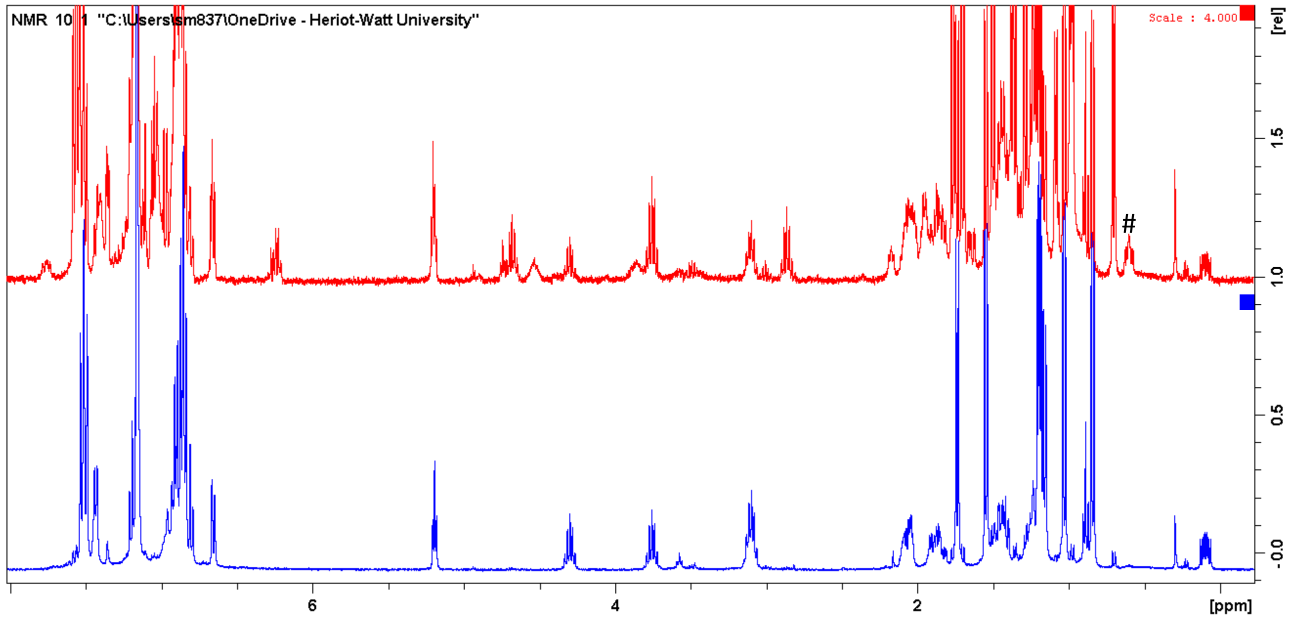

Figure S24. ^1H NMR spectrum (400 MHz, C_6D_6, 298 K) from the thermal decomposition of **8** (red) compared to isolated **14** (blue). The presence of a second, unidentified product is indicated by the characteristic multiplet labelled with #.

3 X-ray crystallography

3.1 Crystallographic details

Single crystals suitable for X-ray diffraction were covered in inert oil and placed under the cold stream of a Bruker D8 Venture diffractometer at 100 K. Exposures were collected using Cu- K_{α} radiation (λ = 1.54178). Indexing, data collection and absorption corrections were performed. The structures were then solved using SHELXT[4] and refined by full-matrix least-squares refinement (SHELXL)[4] interfaced with the programme OLEX2.[5]

Crystals of **12** and **13** were grown from petroleum ether 40-60; single crystals of **10** were grown from hexane. Single crystals of **7** were grown from petroleum ether 40-60 and showed minor disorder in the position of one isopropyl group (0.8:0.2 occupancy modelled using SADI constraints). Single crystals of **8** were grown from pentane and showed positional disorder in the cyclohexene backbone, modelled over two positions (0.51:0.49 occupancy). In this complex, the bond lengths in the ligand do not show longer and shorter C-N and C-C bonds so it was not possible to establish which N donor is an imine and which is an enamide. Instead, it looks like the ligand is disordered so that there is a 50:50 mix of the two donors over both sites, and the H atoms were modelled as such. Crystals of **14** were grown from hexane. Unfortunately, the data quality was not optimal because the nitrogen cold stream of the diffractometer stopped midway during collection, causing the crystal to decompose. The rest of the sample had also decomposed as it had been exposed to oxygen, so we were unable to record any further data. CCDC deposition numbers: 2450673-2450678.

3.2 Additional structures

3.2.1 10

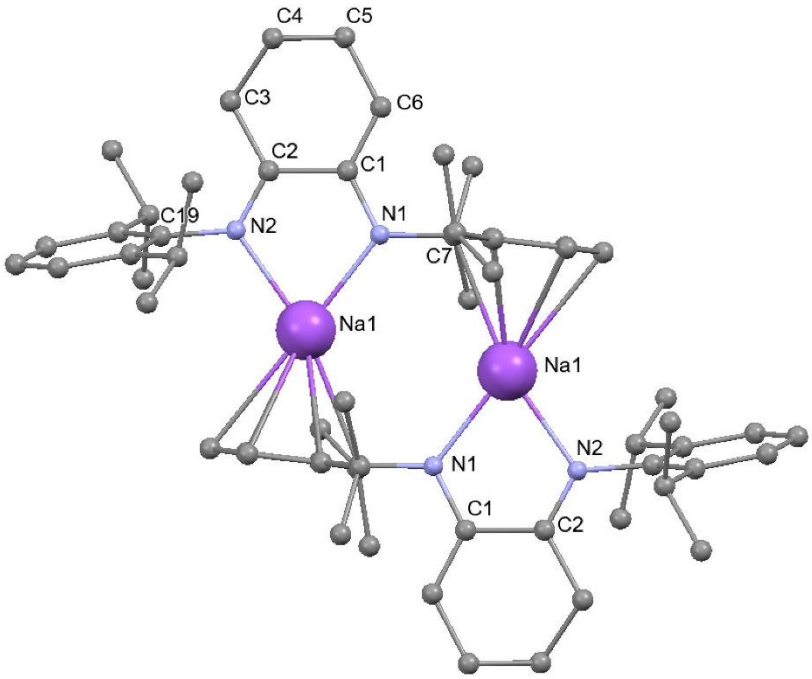

Figure S25. Molecular structure of **10**. All H atoms have been removed for clarity. Na…arene contacts vary from 2.7482(13) to 2.8805(14) Å.

3.2.2 Additional perspective of 14

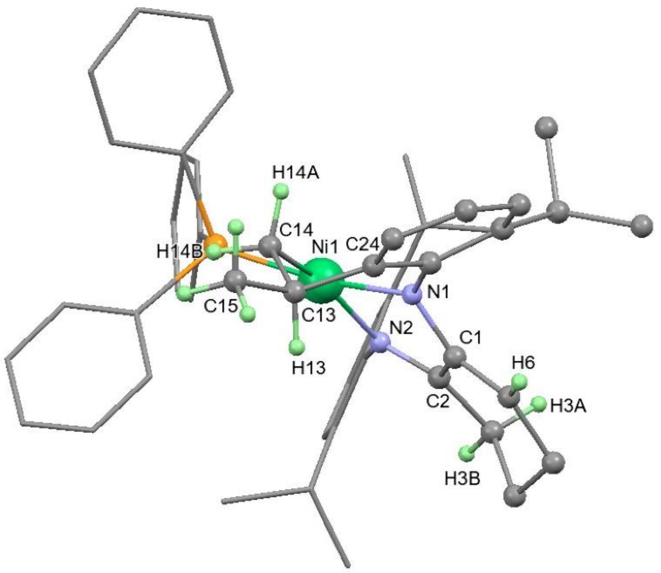

Figure S26. Molecular structure of **14** with significant H atoms labelled.

Table S1. Selected bond lengths (Å) and angles (°).

	Free ligand **12**	Na(ML) **13**	Na(Cy) **10**	NiPh(ML) **7**	NiPh(Cy) **8**	Cyclomet. **14**
N1-C1	1.381(2): enamine	1.369(2): enamide	1.3709(16): enamide	1.362(2): enamide	1.337(2)	1.386(8): enamide
N2-C2	1.273(2): imine	1.289(2): imine	1.2875(16): imine	1.303(3): imine	1.334(2)	1.308(8): imine
**C5-C1**N1 or **C6-C1**N1	1.355(2): enamine	1.375(3): enamide	1.3706(17): enamide	1.390(3): enamide	1.436(2); N.B. C2-C3 = 1.432(2)	1.365(8): enamide
M-N1		2.3048(17)	2.2846(11)	1.9770(16)	1.9787(14)	1.892(5)
M-N2		2.3254(17)	2.3131(11)	1.9769(16)	1.9413(14)	2.011(6)
M-C				1.9098(19)	1.9089(17)	1.957(7)
M-P				2.1676(6)	2.1777(5)	2.173(2)
N-M-N		75.86(6)	72.49(4)	83.37(6)	81.57(6)	81.9(2)
trans N-Ni-P				160.53(5)	162.24(4)	170.28(18)
trans N-Ni-C				172.63(7)	172.19(6)	163.5(3)
Displacement of P away from NiNN plane				0.651	0.604	0.199

3.3 Crystallographic tables of data

Table S2. Additional crystallographic data.

	**12**	**13**	**10**
Empirical formula	$C_{30}H_{42}N_2$	$C_{30}H_{41}N_2Na$	$C_{30}H_{41}N_2Na$
Formula weight	430.65	452.64	452.64
T/K	100.0	100.0	100.0
Crystal system	monoclinic	triclinic	monoclinic
Space group	$P2_1/n$	*P*-1	$P2_1/n$
a/Å	12.8596(2)	10.8949(3)	10.2965(2)
b/Å	13.1817(2)	10.9827(3)	14.7629(3)
c/Å	16.6183(2)	13.6886(4)	18.0717(3)
α/°	90	74.236(2)	90
β/°	107.3660(10)	73.223(2)	99.3880(10)
γ/°	90	63.770(2)	90
Volume/Å^3	2688.59(7)	1386.77(7)	2710.22(9)
Z	4	2	4
ρ_{calc}g/cm^3	1.064	1.084	1.109
μ/mm^1	0.457	0.607	0.621
F(000)	944.0	492.0	984.0
Crystal size/mm^3	0.40 × 0.20 × 0.20	0.18 × 0.06 × 0.04	0.24 × 0.22 × 0.16
Radiation	CuKα (λ = 1.54178)	CuKα (λ = 1.54178)	CuKα (λ = 1.54178)
2Θ range for data collection/°	7.68 to 148.954	6.842 to 144.694	7.774 to 144.48
Index ranges	-16 ≤ h ≤ 15, -16 ≤ k ≤ 16, -20 ≤ l ≤ 20	-13 ≤ h ≤ 13, -13 ≤ k ≤ 12, -16 ≤ l ≤ 16	-12 ≤ h ≤ 12, -17 ≤ k ≤ 18, -22 ≤ l ≤ 22
Reflections collected	86912	24384	72020
Independent reflections	5484 [R_{int} = 0.0364, R_{sigma} = 0.0180]	5449 [R_{int} = 0.0951, R_{sigma} = 0.0702]	5338 [R_{int} = 0.0464, R_{sigma} = 0.0197]
Data/ restraints/ parameters	5484/0/298	5449/0/307	5338/0/306
Goodness-of-fit on F^2	1.060	1.042	1.061
Final R indexes [I>=2σ (I)]	R_1 = 0.0634, $_wR_2$ = 0.1773	R_1 = 0.0525, $_wR_2$ = 0.1270	R_1 = 0.0395, $_wR_2$ = 0.0983
Final R indexes [all data]	R_1 = 0.0661, $_wR_2$ = 0.1798	R_1 = 0.0834, $_wR_2$ = 0.1450	R_1 = 0.0456, $_wR_2$ = 0.1026
Largest diff. peak/hole (e Å^{-3})	1.27/-0.55	0.24/-0.24	0.42/-0.35
CSD deposition numbers	2450673	2450674	2450675

Table S2 continued. Additional crystallographic data.

	**7**	**8**	**14**
Empirical formula	$C_{54}H_{61}N_2NiP$	$C_{54}H_{61}N_2NiP$	$C_{48}H_{55}N_2NiP$
Formula weight	827.72	827.72	749.62
T/K	100.0	100.0	100.0
Crystal system	triclinic	triclinic	monoclinic
Space group	*P*-1	*P*-1	$P2_1/n$
a/Å	11.05020(18)	11.0737(7)	12.1033(13)
b/Å	12.5734(2)	12.5281(10)	15.6179(16)
c/Å	18.4870(4)	18.1740(11)	21.635(3)
α/°	99.0272(11)	97.140(3)	90
β/°	100.2456(11)	100.490(4)	103.455(8)
γ/°	114.5286(6)	115.294(3)	90
Volume/Å^3	2221.80(7)	2182.8(3)	3977.3(8)
Z	2	2	4
ρ_{calc}g/cm^3	1.237	1.259	1.252
μ/mm^1	1.249	1.272	1.341
F(000)	884.0	884.0	1600.0
Crystal size/mm^3	0.12 × 0.06 × 0.02	0.16 × 0.14 × 0.04	0.16 × 0.04 × 0.02
Radiation	CuKα (λ = 1.54178)	CuKα (λ = 1.54178)	CuKα (λ = 1.54178)
2Θ range for data collection/°	5.028 to 144.45	5.078 to 144.784	7.048 to 140.106
Index ranges	-13 ≤ h ≤ 13, -15 ≤ k ≤ 15, -22 ≤ l ≤ 22	-13 ≤ h ≤ 13, -15 ≤ k ≤ 15, -22 ≤ l ≤ 22	-14 ≤ h ≤ 12, -17 ≤ k ≤ 19, -22 ≤ l ≤ 26
Reflections collected	70635	53484	25402
Independent reflections	8706 [R_{int} = 0.0535, R_{sigma} = 0.0319]	8619 [R_{int} = 0.0421, R_{sigma} = 0.0295]	7365 [R_{int} = 0.2535, R_{sigma} = 0.2237]
Data/ restraints/ parameters	8706/6/547	8619/5/564	7365/0/477
Goodness-of-fit on F^2	1.033	1.035	0.944
Final R indexes [I>=2σ (I)]	R_1 = 0.0420, $_wR_2$ = 0.1002	R_1 = 0.0376, $_wR_2$ = 0.0973	R_1 = 0.0798, $_wR_2$ = 0.1613
Final R indexes [all data]	R_1 = 0.0532, $_wR_2$ = 0.1069	R_1 = 0.0417, $_wR_2$ = 0.0998	R_1 = 0.1977, $_wR_2$ = 0.2267
Largest diff. peak/hole (e Å^{-3})	0.98/-0.45	0.45/-0.47	0.39/-0.40
CSD deposition numbers	2450676	2450677	2450678

4 Polymer characterisation

4.1 DSC

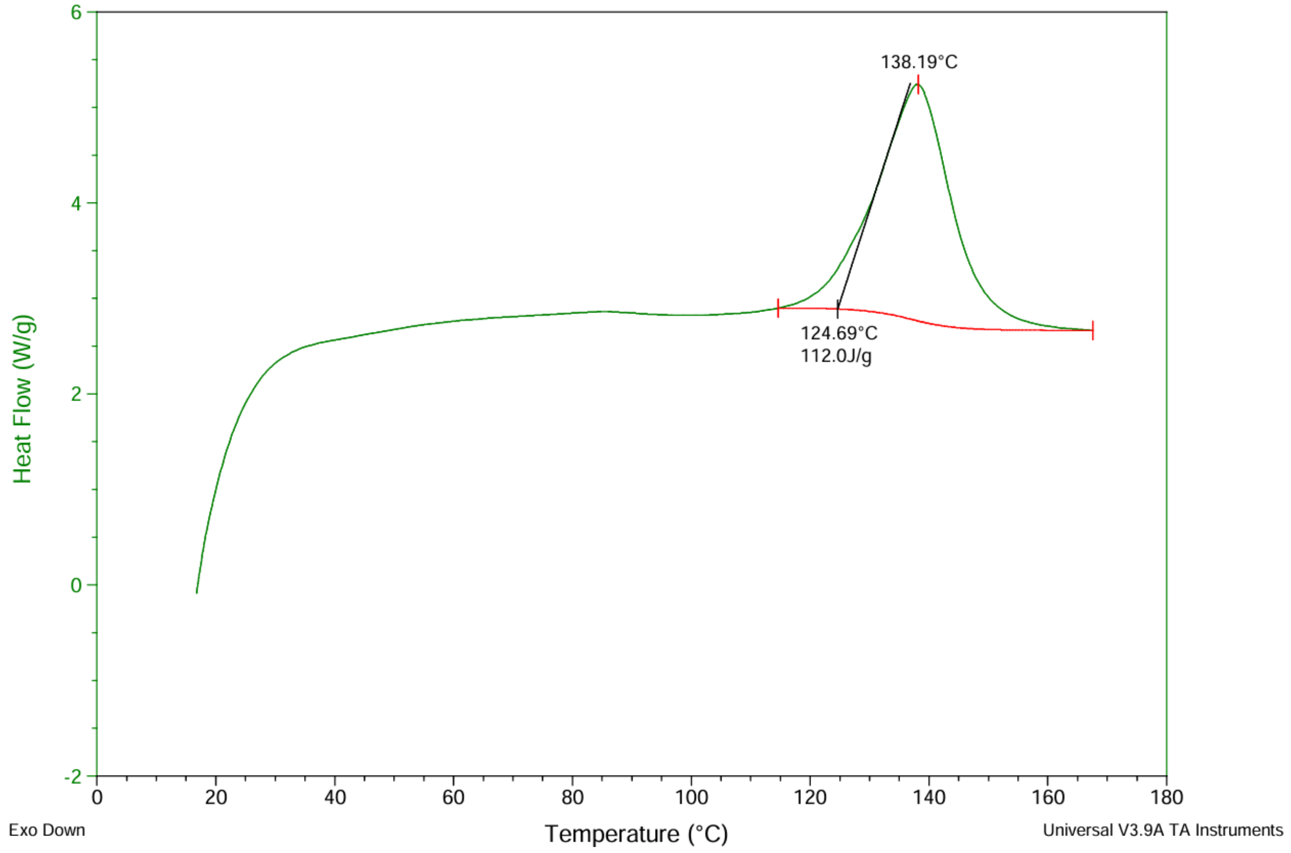

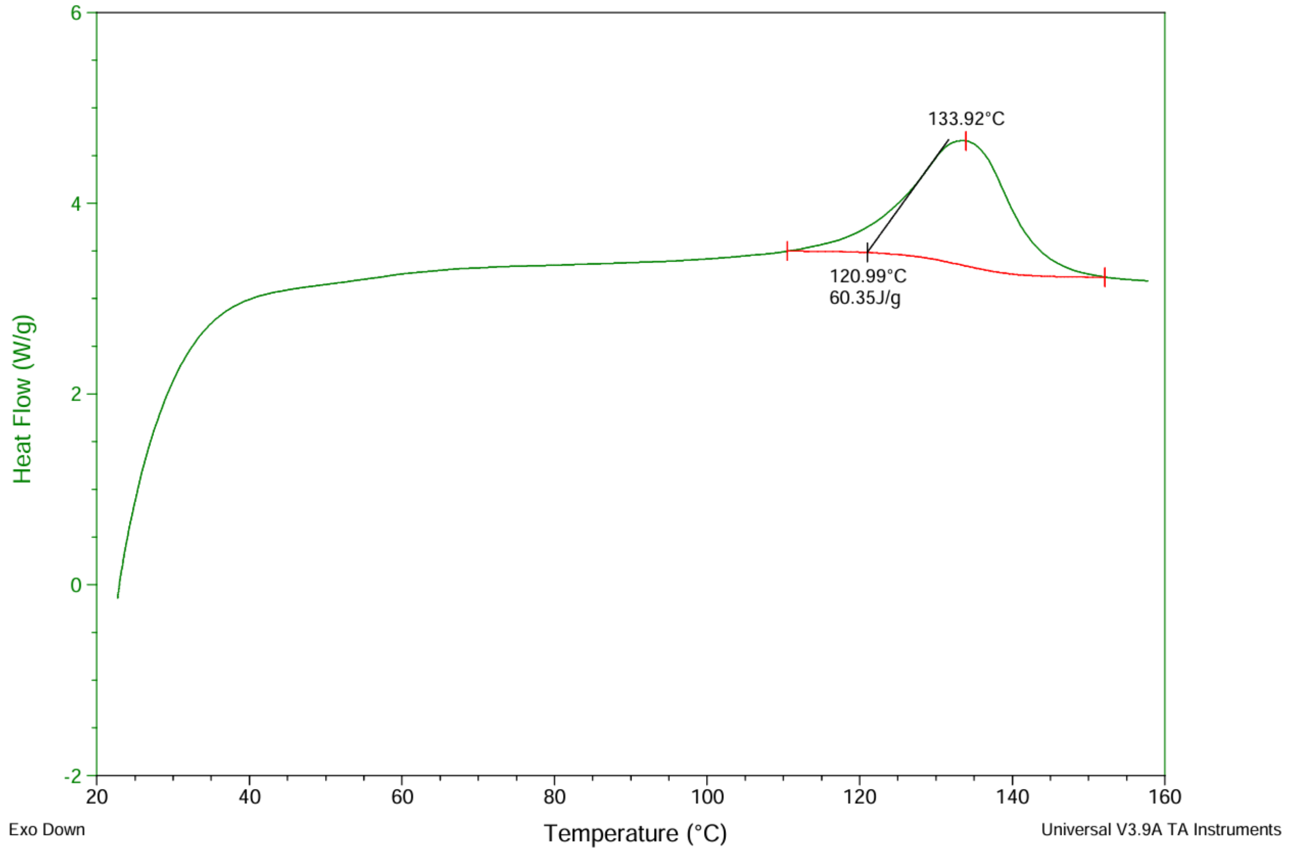

Figure S27. DSC of PE produced under 10 barg ethylene at 0 °C using **7** + $B(C_6F_5)_3$. First run of sample (top) and second run of sample (bottom; sample had crystallised from the melt from the first run).

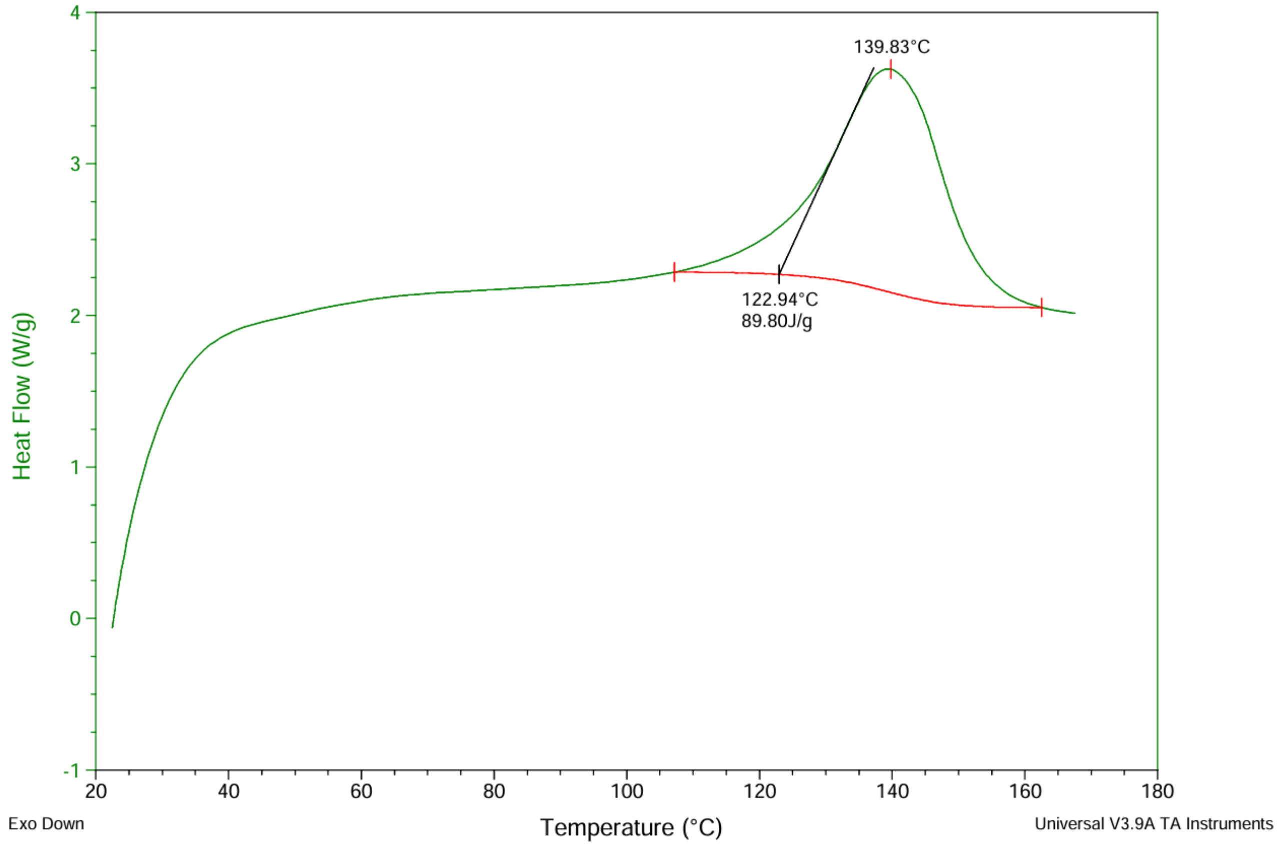

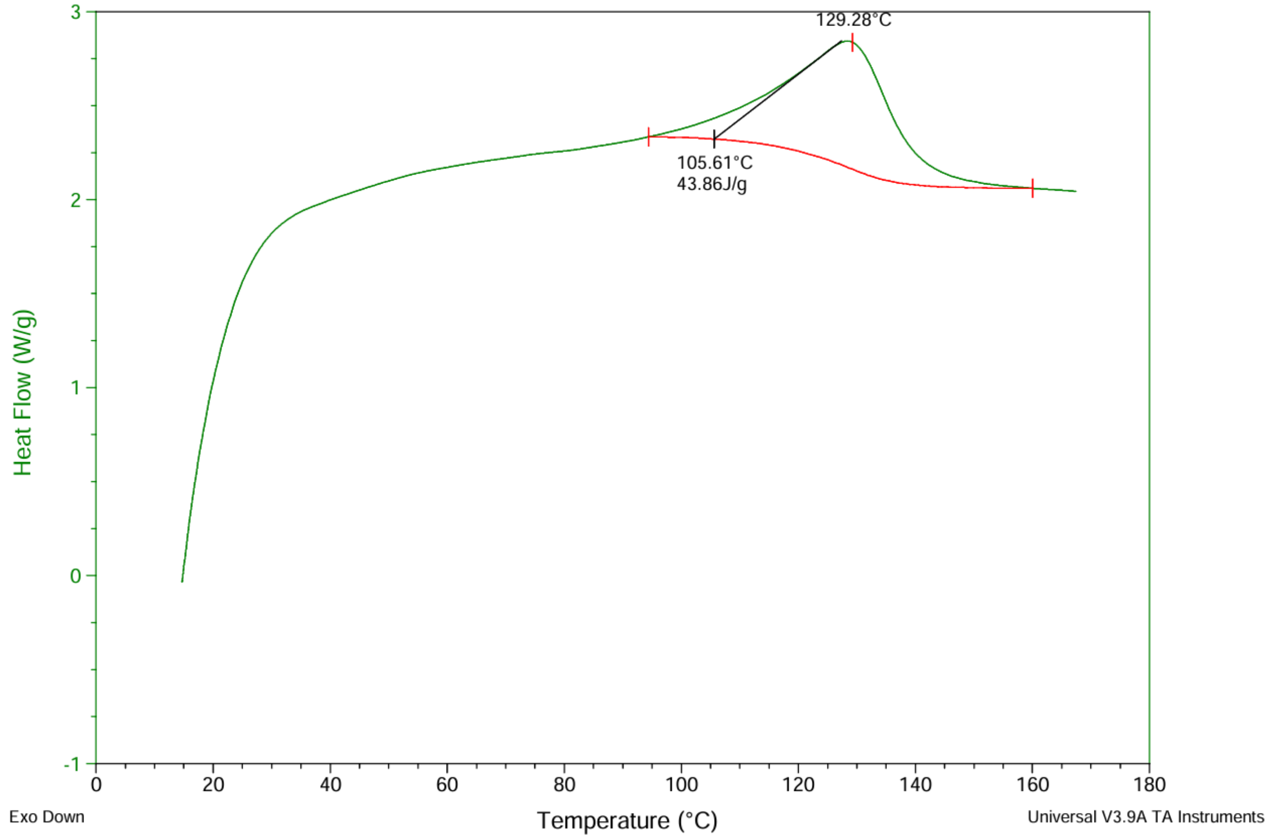

Figure S28. DSC of PE produced under 10 barg ethylene at 0 °C using **8** + $B(C_6F_5)_3$. First run of sample (top) and second run of sample (bottom; sample had crystallised from the melt from the first run).

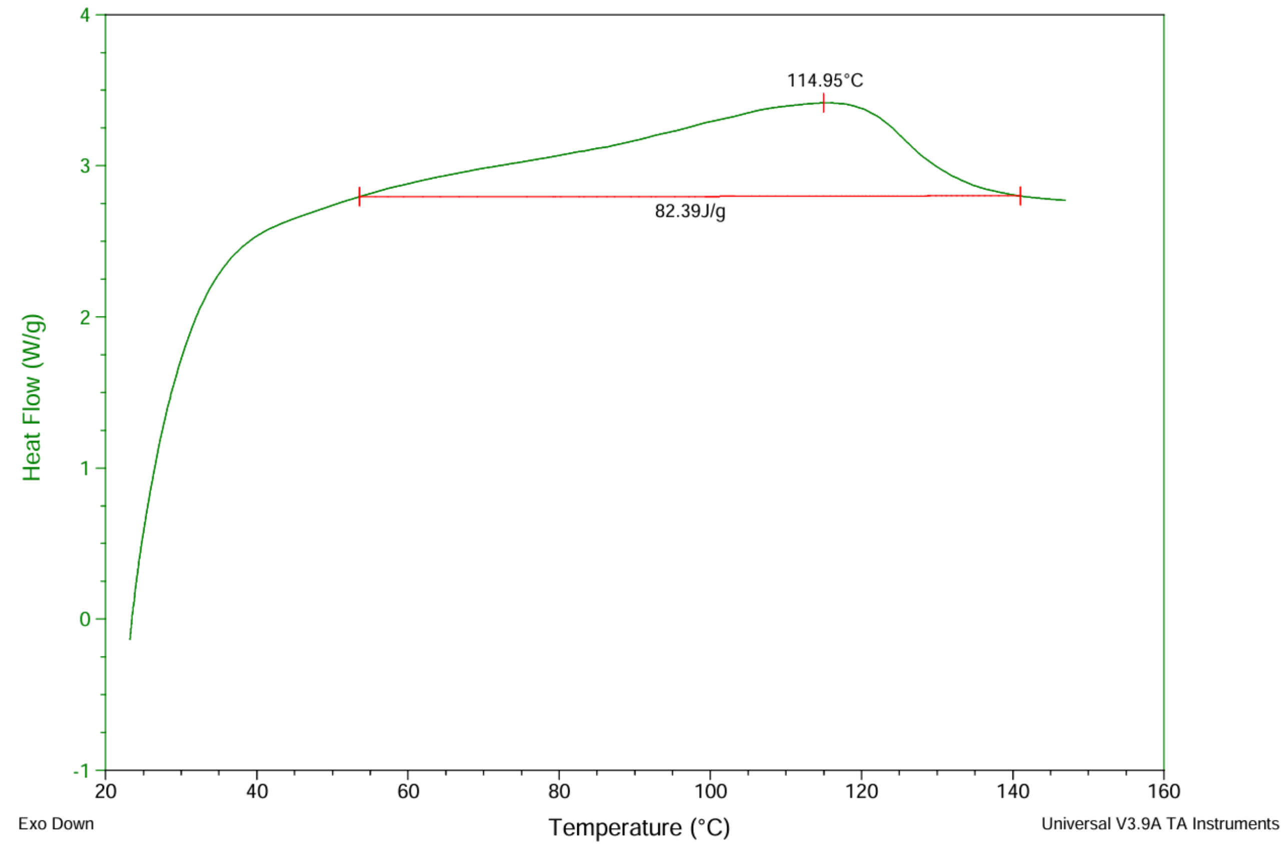

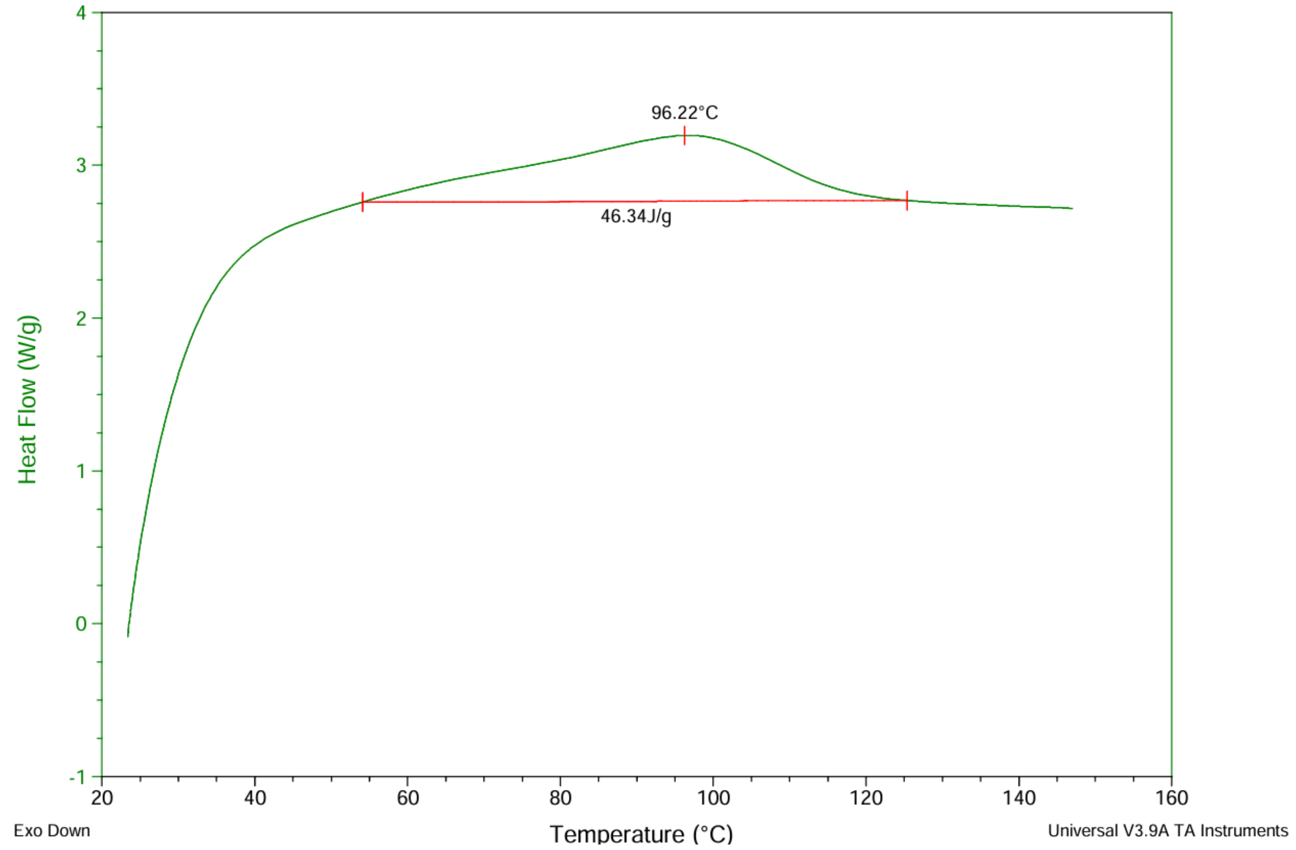

Figure S29. DSC of PE produced under 10 barg ethylene at 25 °C using **8** + $B(C_6F_5)_3$. First run of sample (top) and second run of sample (bottom; sample had crystallised from the melt from the first run).

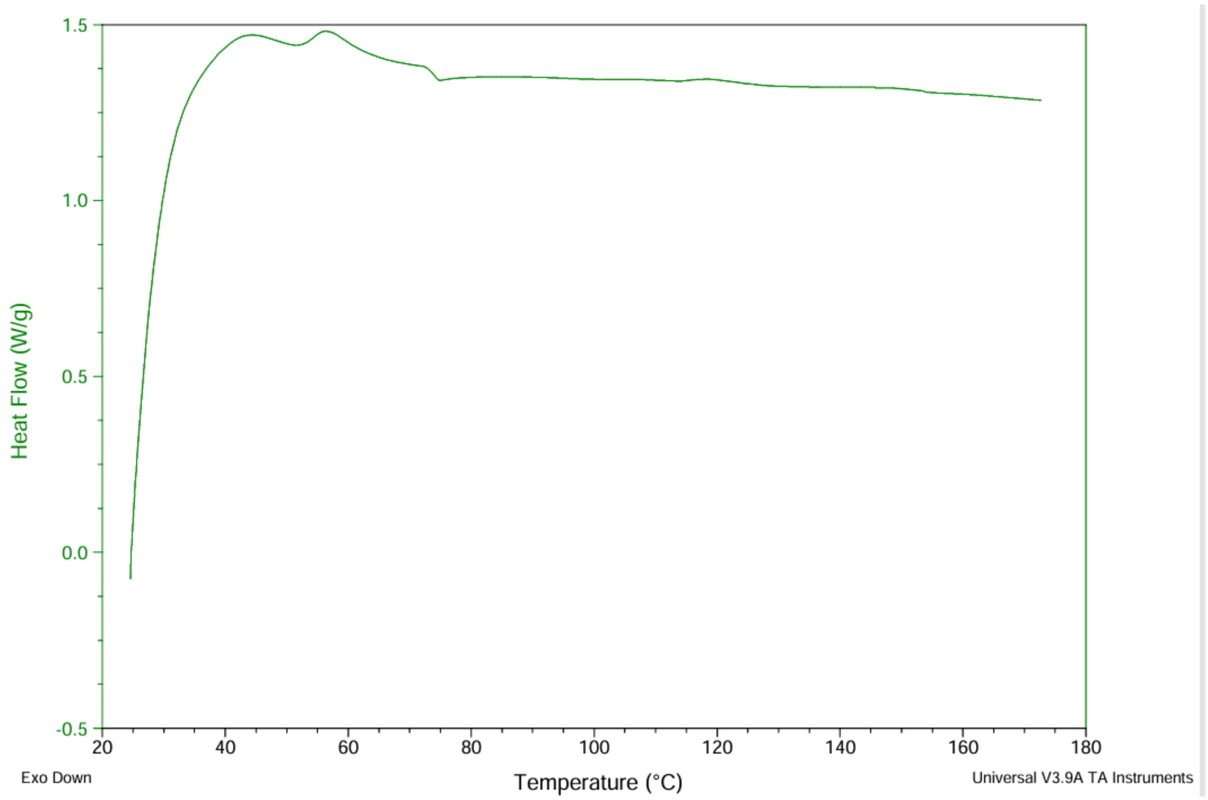

Figure S30. DSC of PE produced under 10 barg ethylene at 70 °C using **8** + $B(C_6F_5)_3$.

4.2 High temperature GPC

Table S3. Mark-Houwink parameters

	**Polystyrene (calibrants)**	**Polyethylene homopolymer (samples)**
Alpha	0.655	0.725
K x 10^{-5} dL/g	19	39

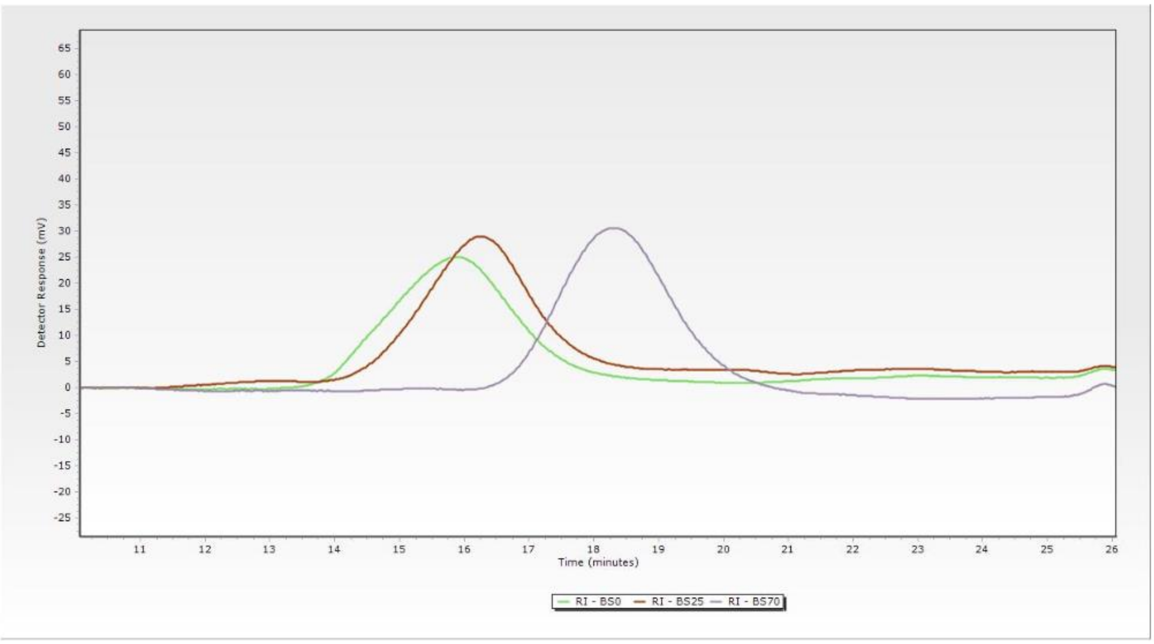

Figure S31. Chromatograms for the PE samples produced by catalyst **8** + $B(C_6F_5)_3$ at 0 °C (green trace), 25 °C (brown trace) and 70 °C (lilac trace).

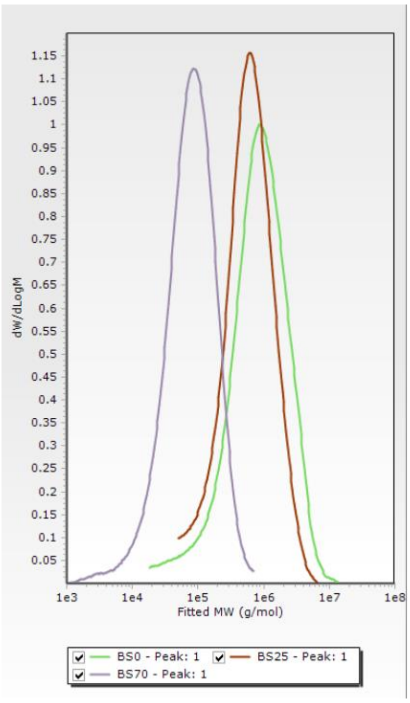

Figure S32. Molecular weight distributions for PE samples produced by catalyst **8** + $B(C_6F_5)_3$ at 0 °C (green trace), 25 °C (brown trace) and 70 °C (lilac trace).

5 References

1. D. P. Gates, S. A. Svejda, E. Oñate, C. M. Killian, L. K. Johnson, P. S. White and M. Brookhart, *Macromolecules*, 2000, **33**, 2320-2334.
2. M. G. Davidson, D. Garcia-Vivo, A. R. Kennedy, R. E. Mulvey and S. D. Robertson, *Chem.-Eur. J.*, 2011, **17**, 3364-3369.
3. A. Zeller, E. Herdtweck and T. Strassner, *Eur. J. Inorg. Chem.*, 2003, **2003**, 1802-1806.
4. E. A. Standley, S. J. Smith, P. Müller and T. F. Jamison, *Organometallics*, 2014, **33**, 2012-2018.
